# Single-molecule sequencing and optical mapping yields an improved genome of woodland strawberry (*Fragaria vesca*) with chromosome-scale contiguity

**DOI:** 10.1093/gigascience/gix124

**Published:** 2017-12-13

**Authors:** Patrick P Edger, Robert VanBuren, Marivi Colle, Thomas J Poorten, Ching Man Wai, Chad E Niederhuth, Elizabeth I Alger, Shujun Ou, Charlotte B Acharya, Jie Wang, Pete Callow, Michael R McKain, Jinghua Shi, Chad Collier, Zhiyong Xiong, Jeffrey P Mower, Janet P Slovin, Timo Hytönen, Ning Jiang, Kevin L Childs, Steven J Knapp

**Affiliations:** 1 Department of Horticulture, Ecology, Evolutionary Biology, and Behavior, Department of Plant Biology, and Center for Genomics Enabled Plant Science, Michigan State University, East Lansing, Michigan, 48823; 2 Ecology, Evolutionary Biology, and Behavior, Department of Plant Biology, and Center for Genomics Enabled Plant Science, Michigan State University, East Lansing, Michigan, 48823; 3 Department of Plant Sciences, University of California - Davis, Davis, California, 95616; 4 Department of Genetics, University of Georgia, Athens, Georgia, 30602; 5 Department of Plant Biology, and Center for Genomics Enabled Plant Science, Michigan State University, East Lansing, Michigan, 48823; 6 Donald Danforth Plant Science Center, St. Louis, Missouri, 63132; 7 Bionano Genomics, San Diego, California, 92121; 8 Potato Engineering and Technology Research Center, Inner Mongolia University, Hohhot, 010021, China; 9 Center for Plant Science Innovation, University of Nebraska, Lincoln, Nebraska, 68588; 10 USDA/ARS, Genetic Improvement of Fruits and Vegetables Laboratory, Beltsville, Maryland, 20705; 11 Department of Agricultural Sciences, Viikki Plant Science Centre, University of Helsinki, Helsinki, 00014, Finland; 12 Center for Genomics Enabled Plant Science, Michigan State University, East Lansing, Michigan, 48823

**Keywords:** *Fragaria vesca*, strawberry, rosaceae, third-generation sequencing, optical map

## Abstract

**Background:**

Although draft genomes are available for most agronomically important plant species, the majority are incomplete, highly fragmented, and often riddled with assembly and scaffolding errors. These assembly issues hinder advances in tool development for functional genomics and systems biology.

**Findings:**

Here we utilized a robust, cost-effective approach to produce high-quality reference genomes. We report a near-complete genome of diploid woodland strawberry (*Fragaria vesca*) using single-molecule real-time sequencing from Pacific Biosciences (PacBio). This assembly has a contig N50 length of ∼7.9 million base pairs (Mb), representing a ∼300-fold improvement of the previous version. The vast majority (>99.8%) of the assembly was anchored to 7 pseudomolecules using 2 sets of optical maps from Bionano Genomics. We obtained ∼24.96 Mb of sequence not present in the previous version of the *F. vesca* genome and produced an improved annotation that includes 1496 new genes. Comparative syntenic analyses uncovered numerous, large-scale scaffolding errors present in each chromosome in the previously published version of the *F. vesca* genome.

**Conclusions:**

Our results highlight the need to improve existing short-read based reference genomes. Furthermore, we demonstrate how genome quality impacts commonly used analyses for addressing both fundamental and applied biological questions.

Eukaryotic genomes, particularly plants, are notoriously difficult to assemble because of issues related to high repeat content, a history of gene and whole-genome duplications, and regions of highly skewed nucleotide composition [1]. The short reads (50–300 bp) generated by second-generation sequencing technologies are often insufficient to resolve complex genomic features and regions. Short reads are unable to span large repetitive regions, resulting in sequence gaps and ambiguities in the assembly graph structures. Despite this known limitation, second-generation sequencing platforms have been used for the majority of genome sequencing projects over the past decade, resulting in a series of unfinished, fragmented draft genome assemblies [2]. For instance, the genome of woodland strawberry (*Fragaria vesca* “*Hawaii-4*”) was assembled using a mixture of different short-read technologies and yielded 16_ _487 contigs in 3263 scaffolds with an N50 length of ∼27 kb [3]. Dense linkage maps were later utilized to split multiple chimeric scaffolds and improve anchoring to the 7 pseudomolecules [4]. However, the *F. vesca* (version 2; V2) genome remains incomplete, with 6.99% gaps, missing megabase-sized regions, and scaffolding errors.

*Fragaria vesca* serves as an important model system for genetic studies for the Rosaceae community, due to its small stature, short generation time, a simple and efficient system for genetic transformation, and an increasing number of genetic resources [5–7]. With more than 2500 described species, Rosaceae is one of the most speciose eudicot families and includes a breadth of important crops (e.g., almonds, apples, apricots, blackberries, cherries, peaches, pears, plums, raspberries, roses, and strawberries) [8]. Furthermore, *F. vesca* is a valuable genetic resource because it is the putative diploid progenitor of the A subgenome of the cultivated octoploid strawberry (*F. x ananassa*) [9]. Strawberries are of major economic importance worldwide, with 373_ _435 hectares planted and 8 114 373 metric tonnes of fruit produced in 2014 [10]. Previous versions of the *F. vesca* genome (V1 and V2) have been used to uncover underlying genetic factors regulating plant and fruit development, seasonal flowering, sex determination, metabolite diversity, and disease resistance [11–16]. A high-quality reference genome for *F. vesca* would further enable family-wide comparative studies and leverage the strengths offered by this model system for both fundamental and applied research.

We aimed to improve the *F. vesca* “*Hawaii-4*” reference genome using a long-read PacBio single-molecule real-time (SMRT) sequencing approach. We generated 2.3 million PacBio reads collectively, spanning 19.4 Gb (×80.8 coverage) with a subread N50 length of 9.2 kb and average length of 8.3 kb (Supplemental Fig. S1; NCBI BioProject ID PRJNA383733). The minimum and maximum read lengths were 3 kb and 72 kb, respectively. The raw PacBio reads were error-corrected and assembled using the Canu [17] assembler, followed by 2 rounds of polishing with Quiver [18]. High-coverage (∼×40) Illumina data were aligned to the PacBio assembly, and residual errors were corrected using Pilon [19]. After removing the complete chloroplast and mitochondrial genomes, the final assembly spanned 219 Mb across 61 contigs with an N50 length of 7.9 Mb. Half of the assembly is contained in the largest 9 contigs, including 5 that exceed 10 Mb. The assembly graph is relatively simple with few ambiguities, excluding a small cluster of 5 contigs corresponding to rRNA gene arrays from the nucleolar organizer region (Supplemental Fig. S2). This represents a ∼300-fold improvement in contiguity compared with the Illumina and 454-based *F. vesca* V1 assembly [3].

The PacBio-based contigs were anchored into a chromosome-scale assembly using a 2-enzyme BioNano Genmomics optical map. Contigs were scaffolded first using the BsqQI map, and this hybrid assembly was used as a reference for the BssSI map. Incongruences between the genome assembly and optical map were screened using a hybrid scaffold algorithm from BioNano Genomics and manual curation, which resulted in a total of 7 cuts made to input contigs and a single cut made to the optical map. Furthermore, Structural Variation detection between the BspQI assembly and the final output detected no major conflicts within the optical map resolution range. The combined BioNano and PacBio assembly spans 220.8 Mb across 31 scaffolds with an N50 length of 36.1 Mb, with 99.8% of the assembly captured in 9 scaffolds (Supplemental Table S1). Five of the 7 *F. vesca* chromosomes are complete, and 2 chromosomes were assembled into chromosome arms. The 2 pairs of chromosome arms were anchored using support from genetic maps [3]. The PacBio and BioNano assembly (hereafter referred to as *F. vesca* V4) captures ∼24.96 Mb of additional sequences with significant improvements in contiguity. The average gap size in the V2 assembly is >1 kb. Nearly all of these gaps, in total, ∼17 Mb of missing sequence (i.e., Ns), in the V2 assembly were filled. It's difficult to assess the exact number of gaps that were filled due to the drastic improvement of the V4 assembly. A total of 37 gaps remain in the V4 assembly after BNG hybrid scaffolding, including 23 kb of missing sequence, with an average gap size of 621 bp. These gaps likely correspond to highly complex, repetitive regions that are difficult to assemble. These gaps may also include unanchored sequences that had no label sites in the BNG optical maps.

*F. vesca* V4 has 9 terminal telomere tracks with sequence and genome map support (Fig. 1, Supplemental Fig. S3), suggesting that the assembly is largely complete. Tandem arrays of centromeric repeats with monomeric lengths of 140, 143, and 147 bp were found in all 7 chromosomes, consistent with previous findings [3]. *F. vesca* V4 contains 3 nucleolus organizer regions (NORs) at the beginning of Fvb1 and Fvb7 and at the end of Fvb5, consistent with previous cytological observations [20]. NOR rRNA arrays are complete on Fvb1 and Fvb5, but fragmented on Fvb7, based on sequence and genome map support. The 5S rRNA array is located 5 Mb upstream of the NOR on Fvb7 (Supplemental Fig. S4).

**Figure 1:**
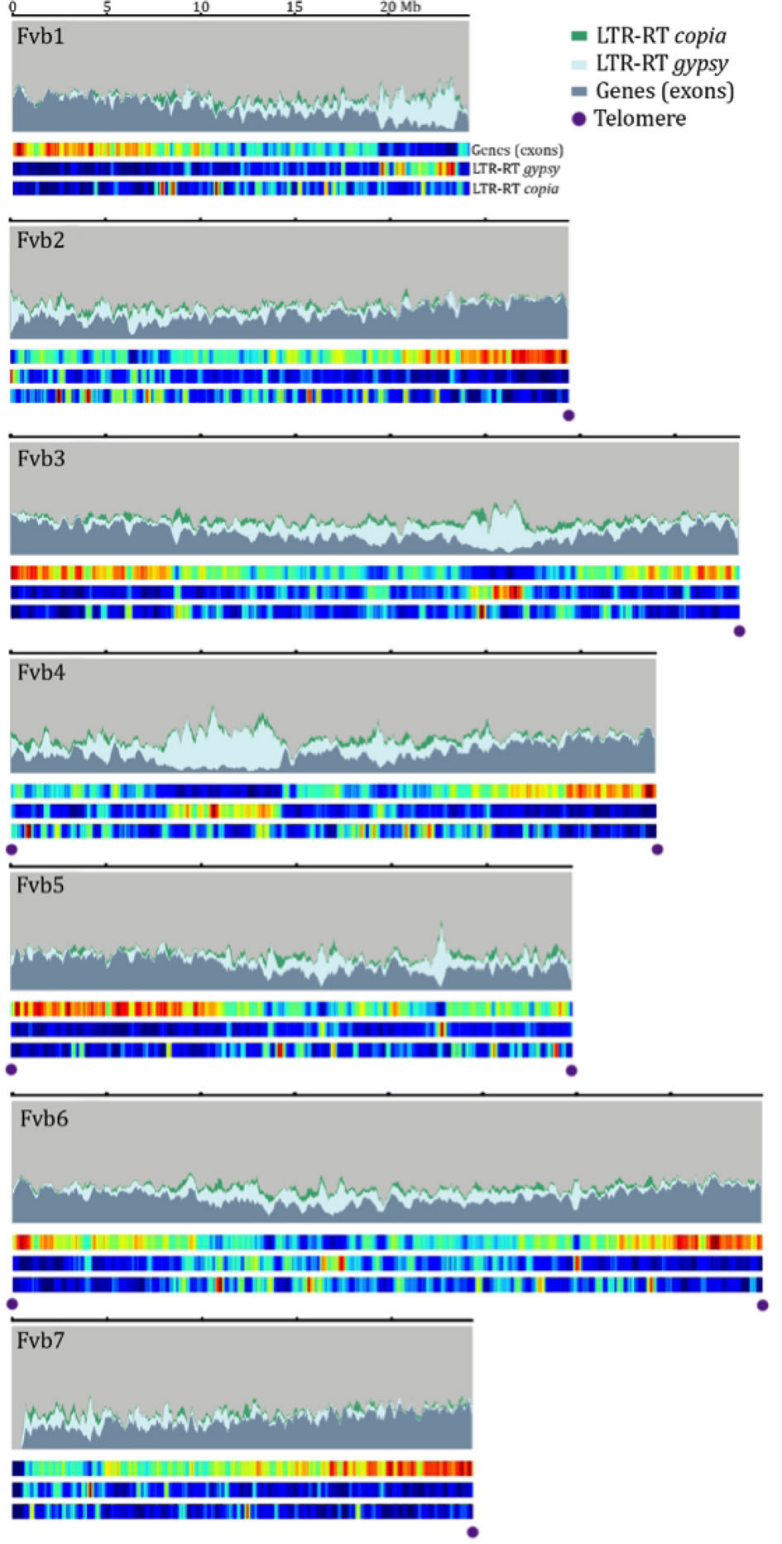
Chromosome landscapes of the *F. vesca* V4 genome. The distribution of genes and long terminal repeat retrotransposons (LTR-RTs) are plotted for each of the 7 chromosomes. Heatmaps reflect the distribution of elements, with blue indicating the lowest abundance and red signifying high abundance. Plots were generated with a sliding window of 50 kb, with a 10-kb shift across each chromosome. Terminal telomeric repeat arrays are denoted in purple.

A whole-genome comparison of *F. vesca* V4 to V2 [4] uncovered numerous, large-scale scaffolding errors made in each of the chromosomes in the previous version (Fig. 2). The overall quality of the *F. vesca* V4 assembly, compared with V2, is also supported by the distribution pattern of DNA methylation across chromosomes (Supplemental Fig. S5). These types of errors considerably hinder various genomic analyses, including fine-mapping genes underlying traits [21] and identifying structural variants via comparative genomics. Here we demonstrate the superior quality of *F. vesca* V4 by making comparisons with a high-density linkage map of *Fragaria iinuma*e [22], which is another putative diploid progenitor species of the cultivated octoploid strawberry. The total number of collinear markers against the *F. iinumae* genetic map increased by more than 10% using *F. vesca* V4, compared with V2, and identified a distinctive chromosomal inversion between the 2 species near the pericentromeric region on chromosome 3 (Supplemental Fig. S6, Supplemental Table S2, Table S1).

**Figure 2:**
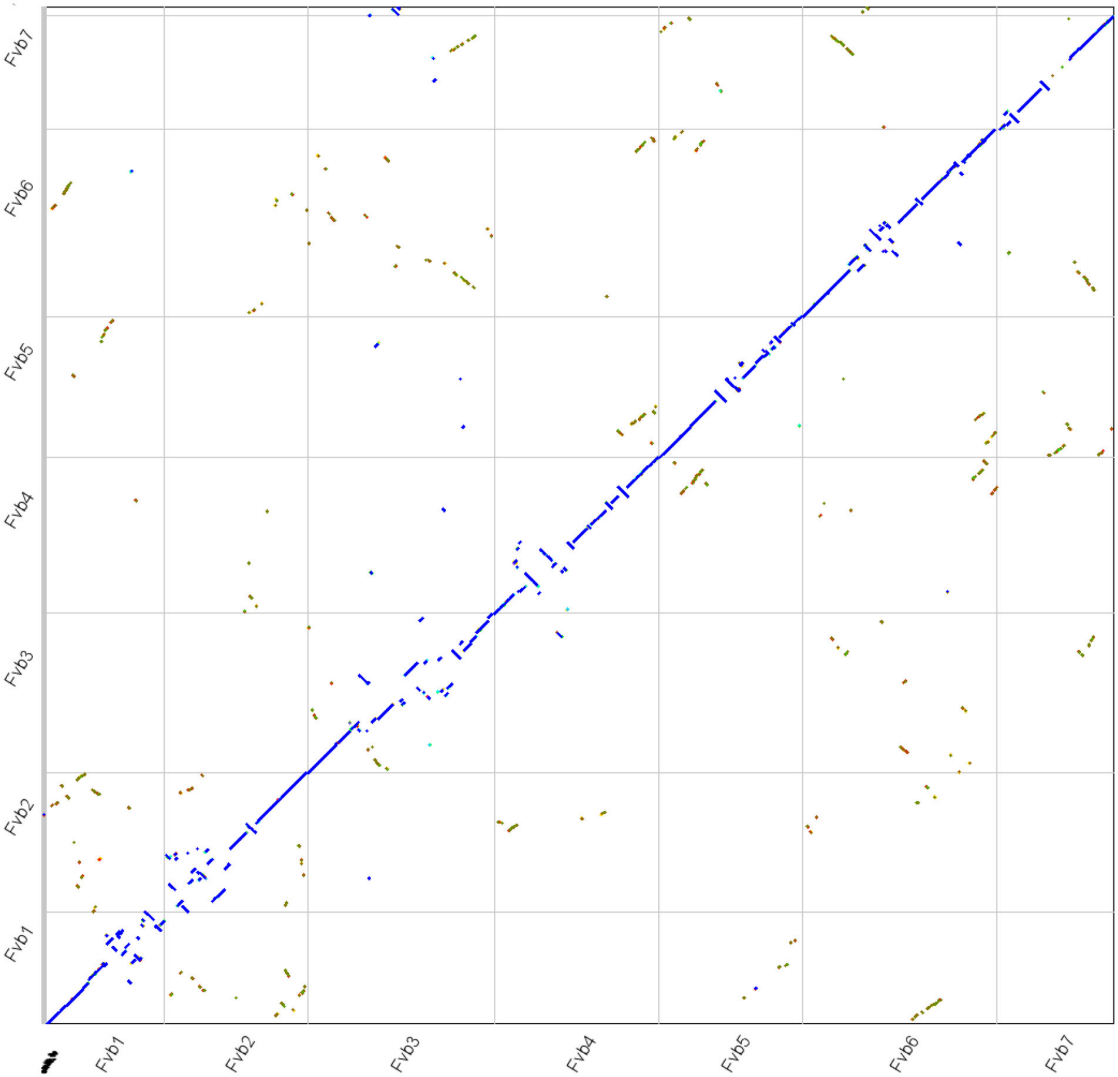
Macrosyntenic comparison of the V2 and V4 *F. vesca* assemblies. Syntenic gene pairs between V4 (x-axis) and V2 (y-axis) of *F. vesca* were identified by DAGChainer [44], sorted by chromosome (Fvb1-7), and colored based on their synonymous substitution rate, as calculated by CodeML [45] using SynMap within CoGe [46]. Syntenic “orthologous” regions are colored in blue, and duplicated genes retained from a whole-genome triplication event (At-gamma [47]) in other colors. Regions that were misassembled and incorrectly scaffolded in *F. vesca* V2 are identified by negatively sloped and repositioned lines.

Although the quality of previous annotations of the *F. vesca* genome [3, 23] is comparable with other annotations of short-read assemblies, they are, unavoidably, incomplete and fragmented, resulting in errors in gene identification and gene number predictions [24]. Thus, despite the increasing volume of transcript and protein sequence information generated from various experimental studies, the task of improving genome annotation of such genomes remains a major challenge. Using the MAKER-P annotation pipeline (MAKER, RRID:SCR_005309) [25], publicly available transcriptome data of *F. vesca*, and protein sequences from *Arabidopsis thaliana* and the UniprotKB database as evidence, we identified 28_ _588 gene models in *F. vesca* V4, of which 70% have a known Pfam domain and 27_ _491 are supported by RNA-seq data. The mean length of the predicted genes is 1475 bp (Supplemental Table S3). Repetitive elements were annotated, including long terminal repeat retrotransposons (LTR-RTs; e.g., *gypsy* and *copia*) (Fig. 1), non-LTR retrotransposons, and DNA transposons, using RepeatModeler (RepeatModeler, RRID:SCR_015027) [26], MITE_Hunter [27], and LTR_retriever [28]. Most repetitive elements are unassembled, incomplete, or collapsed in short-read-based reference genomes, which results in the underestimation of the repeat content of most eukaryotic genomes [29]. The improvement in genome quality of *F. vesca* V4 permitted the identification of additional LTR-RTs (Supplemental Table S4). Furthermore, an analysis of the insertion times of each LTR-RT indicates that there were 2 major LTR-RT bursts; approximately 1.8 and 1.2 million years before present (Supplemental Fig. S7). Organellar genomes from the plastid and mitochondrion were also annotated and verified for completeness (Supplemental Figs S8 and S9).

The Benchmarking Universal Single-Copy Orthologs V2 (BUSCO, RRID:SCR_015008) [30] method was used to estimate the completeness of genome assembly and quality of gene annotation of *F. vesca* V4. The majority (95%) of the 1440 core genes in the embryophyta dataset were identified in the annotation, which is supportive of a high-quality assembly and annotation similar to other high-quality grade genomes [31–33]. The overall quality of the annotation is further supported by the distribution of DNA methylation across the gene bodies (Fig. 3). The *F. vesca* V4 annotation shows much sharper distribution patterns, especially in the CG context, and lower CHG and CHH (where H = A, T, or C) methylation in the gene bodies. These patterns are expected for annotations that are more accurate and contain fewer mis-annotations (e.g., pseudogenes, transposons, etc.). Additionally, *F. vesca* V4 contains 1496 newly predicted gene models, with a mean length of 1505 bp, that were not present in all previous versions of the annotation [3, 23]. The vast majority of these new genes (1463 total) are expressed in different fruit tissues and developmental stages (Fig. 4; Table S2). These newly identified genes either resided within the gaps in the V2 assembly or were collapsed tandem duplicates in the previous V1 assembly. Thus, previous expression studies may have missed key genes controlling fruit development and maturation in *F. vesca* [34, 35]. Of the new genes in *F. vesca* V4, 810 genes did not show similarity at the protein level (query length < 30%, E = 10^−10^) to any paralogs in the V2 genome but exhibit unique expression patterns (Fig. 4). We also identified significantly more tandemly duplicated genes and larger tandem arrays in *F. vesca* V4 (Supplemental Fig. S10). Long-read single molecule sequencing approaches have been shown to better resolve tandemly repeated copies [36–38]. The identification of tandemly duplicated genes is important as such genes are known to be highly enriched for both abiotic and biotic stress-related functions [39]. For example, many important plant defense genes, including nucleotide-binding site leucine-rich repeat (*NBS-LRR*) [40] and cytochrome p450s (*CYPs*) [41], are tandemly duplicated and exhibit high levels of copy number variation within a species.

**Figure 3:**
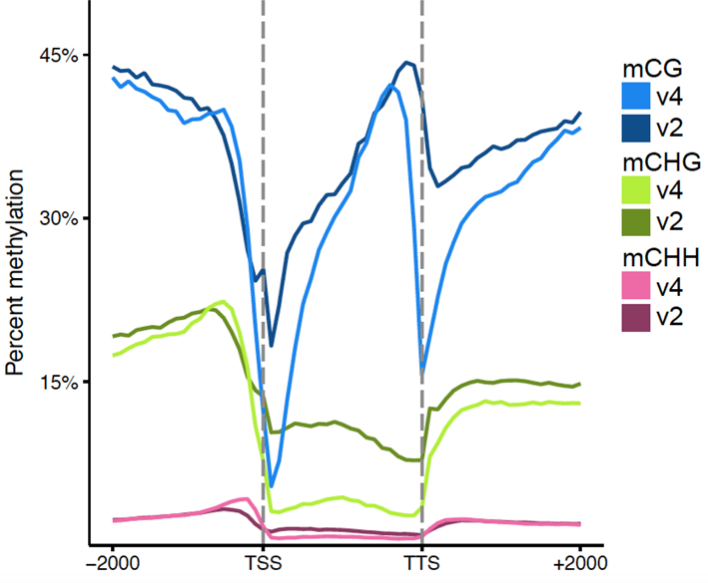
Distribution of gene body methylation in the V2 and V4 *F. vesca* assemblies. This plot shows the average DNA methylation patterns (CG = blue, CHG = green, CHH = red; H = A, T, or C) across all genes in the V2 (darker colors) and V4 (lighter colors) assemblies. The x-axis shows the transcription start sites (TSS; left dashed line) and the transcription termination sites (TTS; right dashed line), plus +/- 2000 bp from each gene.

**Figure 4:**
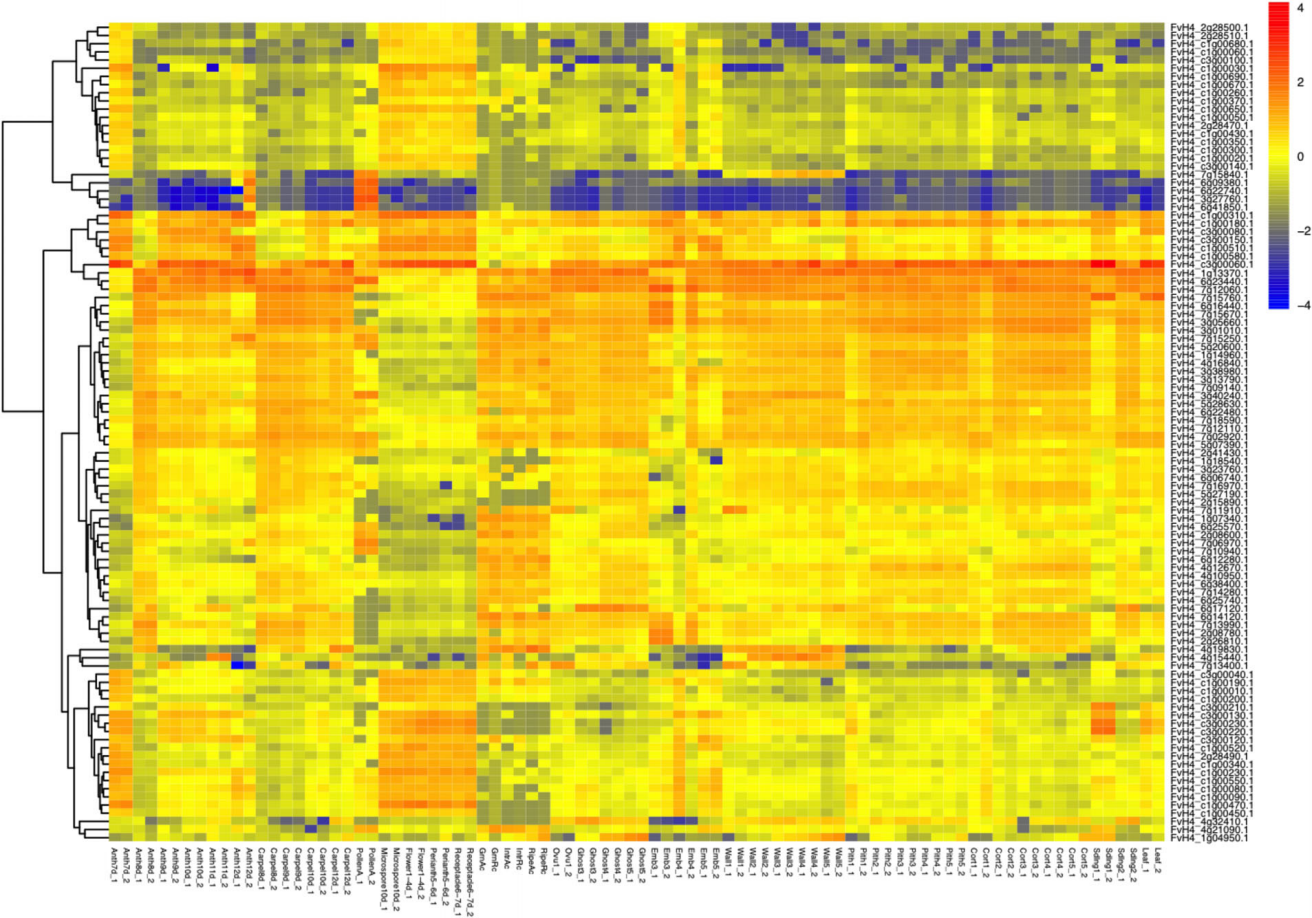
Expression patterns of newly annotated genes across diverse tissue types. Heatmap consists of a random subset of 100 genes from the unique 810 newly identified genes in the *F. vesca* V4 assembly, across 22 tissue types at different developmental stages. Two biological replicates were sequenced per tissue, with the exception of 6 with only 1 biological replicate each (Table S2). Blue indicates the lowest expression, and red signifies the highest expression abundance. Gene expression level was calculated based on reads per kilobase of transcript per million mapped reads (RPKM) and visualized through heatmap analysis using variance-stabilized transformed values on a log2 scale.

Here we present one of the most complete and contiguous plant genomes assembled to date. The average published plant genome is highly fragmented, with a contig N50 length of roughly 50 kb [2], compared with ∼7.9 Mb for *F. vesca* V4. The *F. vesca* V4 genome has the third best contig N50 of any angiosperm sequenced to date, after only *Arabidopsis thaliana* [42] and rice (*Oryza sativa*) [43]. It is important to note that the total cost for a PacBio-sequenced and BioNano Genomics genome is a very small fraction of the cost compared with these Sanger-era genomes [31]. Our genomic analyses, which included direct comparisons with previously published versions (V1 and V2) of the same genotype [3, 4, 23], highlight the need to improve existing short-read-based reference genomes. The approach used here, combining long-read sequencing and optical maps, corrects mis-assembly and scaffolding errors commonly found in short-read-based genomes, which dramatically impact the results in genetic mapping (Supplemental Fig. S6), methylation (Fig. 3), and gene expression studies (Fig. 4).

## Availability of supporting data

The genome assembly, annotations, and other supporting data are available via the *GigaScience* database, *Giga*DB [48]. The *F. vesca* V4 assembly and annotation will also be made publicly available on the *Genome Database for Rosaceae* [49] and the *CyVerse CoGe* platform [50]. The raw sequence data have been deposited in the Short Read Archive under NCBI BioProject ID PRJNA383733.

## Additional files

Additional file: H4_TableS1.xlsx

Additional file: H4_TableS2.xlsx

Additional file: Supplement-H4GenomePaper_Final3.docx

## Competing interests

The authors declare that they have no competing interests.

## Author contributions

P.P.E., R.V., and S.J.K. designed research; P.P.E., R.V., M.C., T.J.P., C.M.W., C.E.N., E.A., S.O., C.B.A., J.W., P.C., M.R.M., J.S., C.C., Z.X., J.P.M., J.P.S., T.H., N.J., K.L.C., and S.J.K. performed research and/or analyzed data; and P.P.E., R.V., M.C., E.A., and S.J.K wrote the paper. All authors reviewed the manuscript.

## Abbreviations

bp: base pair; BUSCO: Benchmarking Universal Single-Copy Orthologs; kb: kilo base; LTR-RT: long terminal repeat retrotransposons; Mb: mega base; NOR: nucleolus organizer regions; rRNA: ribosomal RNA; TE: transposable element. 

## Supplementary Material

GIGA-D-17-00135_Original_Submission.pdf

GIGA-D-17-00135_Revision_1.pdf

GIGA-D-17-00135_Revision_2.pdf

Response_to_Reviewer_Comments_Original_Submission.pdf

Response_to_Reviewer_Comments_Revision_1.pdf

Reviewer_1_Report_(Original_Submission) -- Yuxuan Yuan 26 Jun 2017 Reviewed

Reviewer_1_Report_(Revision_1) -- Yuxuan Yuan 18 Jul 2017 Reviewed

Reviewer_2_Report_(Original_Submission) -- Shiguo Zhou 30 Jun 2017 Reviewed

Supplemental data
