# Supplementary material for: Single-molecule sequencing and optical mapping yields an improved genome of woodland strawberry (Fragaria vesca) with chromosome-scale contiguity
Source: Gigascience. 2017 Dec 13;7(2):gix124. doi: 10.1093/gigascience/gix124 (PMC5801600; doi:10.1093/gigascience/gix124)
Supplement: GIGA-D-17-00135_Revision_1.pdf [file gix124_giga-d-17-00135_revision_1.pdf]

GigaScience

# Single-molecule sequencing and optical mapping yields an improved genome of woodland strawberry (Fragaria vesca) with chromosome-scale contiguity

--Manuscript Draft--

| | |
|---|---|
| Manuscript Number: | GIGA-D-17-00135R1 |
| Full Title: | Single-molecule sequencing and optical mapping yields an improved genome of woodland strawberry (Fragaria vesca) with chromosome-scale contiguity |
| Article Type: | Data Note |
| Funding Information: | USDA-HATCH (1009804) — Dr Patrick Edger |
| Abstract: | Although draft genomes are available for most agronomically important plant species, the majority are incomplete, highly fragmented, and often riddled with assembly and scaffolding errors. These assembly issues hinder advances in tool development for functional genomics and systems biology. Here we utilized a robust, cost-effective approach to produce high-quality reference genomes. We report a near-complete genome of diploid woodland strawberry (Fragaria vesca) using single-molecule real-time sequencing from Pacific Biosciences (PacBio). This assembly has a contig N50 length of ~7.9 Mb, representing a ~300 fold improvement of the previous version. The vast majority (>99.8%) of the assembly was anchored to seven pseudomolecules using two sets of optical maps from Bionano Genomics. We obtained ~24.96 million base pairs (Mb) of sequence not present in the previous version of the F. vesca genome and produced an improved annotation that includes 1,496 new genes. Comparative syntenic analyses uncovered numerous, large-scale scaffolding errors present in each chromosome in the previously published version of the F. vesca genome. Our results highlight the need to improve existing short-read based reference genomes. Furthermore, we demonstrate how genome quality impacts commonly used analyses for addressing both fundamental and applied biological questions. |
| Corresponding Author: | Patrick Edger<br>Michigan State University<br>UNITED STATES |
| Corresponding Author Secondary Information: | |
| Corresponding Author's Institution: | Michigan State University |
| Corresponding Author's Secondary Institution: | |
| First Author: | Patrick Edger |
| First Author Secondary Information: | |
| Order of Authors: | Patrick Edger |
| | Robert VanBuren |
| | Marivi Colle |
| | Thomas Poorten |
| | Ching Man Wai |
| | Chad Niederhuth |
| | Elizabeth I Alger |
| | Shujun Ou |
| | Charlotte Acharya |
| | Jie Wang |
| | Pete Callow |
| | |

Powered by Editorial Manager® and ProduXion Manager® from Aries Systems Corporation

| | |
|---|---|
| | Michael McKain |
| | Jinghua Shi |
| | Chad Collier |
| | Zhiyong Xiong |
| | Jeffrey Mower |
| | Janet Slovin |
| | Timo Hytönen |
| | Ning Jiang |
| | Kevin Childs |
| | Steven Knapp |
| **Order of Authors Secondary Information:** | |
| **Response to Reviewers:** | Reviewer #1: Edger P and colleagues present an improved Fragaria vesca genome assembly using PacBio long read sequencing and BioNano optical mapping. In their report, they claimed that their new assembly was one of the most complete and contiguous plant genome assemblies, which is interesting and impressive. In their studies, they compared the new assembly (V4) with the old V2 short read assembly and claimed that they had improved the Fragaria vesca genome assembly to a 'platinum' standard. However, to publish on GigaScience, I think they may address the concerns below:<br><br>Response: Thank you for your comments and suggestions. We believe that having addressed these comments helped strengthen the overall quality of the manuscript.<br><br>Major:<br>1. How do authors define 'platinum' quality reference genomes? In what stage can a draft reference genome be called a 'platinum' quality reference genome?<br><br>Response: We have changed all instances of ‘platinum quality’ to ‘high-quality’.<br><br>2. What was the coverage of the raw 'BspQI' BioNano maps and the coverage of the raw 'BssSI' maps?  It will be good to give a statistical report of the raw BioNano maps.<br><br>Response: We agree and have added a new table with these details to the supplement.<br><br>3. In the manuscript, authors using the 'BspQI' maps completed the first-round hybrid scaffolding and 'BssSI' maps did the second-round hybrid scaffolding. How about changing the enzyme order to perform 'BssSI' hybrid scaffolding first and then the 'BspQI' hybrid scaffolding? Will this change the result and which method gives a better assembly?<br><br>Response: The results will be similar no matter which enzyme map is used first, as long as both enzyme produced a high-quality assembly (which is the case here; see the new table in the supplement). Furthermore, the restriction site distribution pattern largely matches between the hybrid scaffolds and the contigs, except for the few instances discussed below. BspQI was chosen for the first round because its assembly was more complete than BssSI (250Mb vs. 214 Mb), and its contiguity was better (2.5 Mb vs. 1.3 Mb).<br><br>4. In the first-round BNG hybrid assembly, authors selected the parameter settings as 'cut contig at conflict in BNG maps' and 'cut contig at conflict in NGS sequences'. Shouldn't authors keep the BNG maps and cut the NGS sequences when conflicts occur, as BNG single molecule maps are much longer than the PacBio single reads?<br><br>Response: When conflicts occur, the hybrid scaffold algorithm checks the chimeric quality score of the bionano assembly at the break point. If the score >=30, the |

Powered by Editorial Manager® and ProduXion Manager® from Aries Systems Corporation

confidence of single long molecule support of the bionano assembly is normally very strong, and NGS assembly will be cut at this break point. If the score is <30, there might be a chance that the bionano assembly is chimeric, then the BNG map will be cut. Here, for F. vesca V4, there were 7 cuts made to the contigs and 1 cut made to the BNG map. We manually checked all cut sites, and they all looked quite convincing based on our experience.

5. I noticed that there were still some conflicts between the new V4 assembly and BNG maps. It would be good to validate the BNG hybrid assembly or the final V4 assembly using optical mapping to check how many conflicts unsolved using such as BioNano SV detection (here SV regions should be misassembled regions or conflict regions). What solutions will authors use to solve those detected conflicts?

Response: We (coauthors at BNG) ran Structural Variation (SV) detection between the BspQI assembly and the final V4. There were no major conflicts in the calls with reasonable confidence. 60 deletion calls and 89 insertion calls, all within 150bp, which is less than the optical map resolution range. Our pseudomolecules are also congruent with the published genetic map which we used to anchor the two sets of chromosome arms.

6. How many unknown sequences (gaps) obtained after BNG hybrid scaffolding? How many gaps have been filled in V4 compared to V2? What's the average size of those unfilled gaps? What caused those unfilled gaps?

Response: The F. vesca assembly (Shulaevet al. 2011) has 15,798 contigs with an N50 of 27 kb. The average gap size in the V2 assembly is 1,076 bp. Our assembly has 61 contigs with a contig N50 of 7.9 Mb (before bionano anchoring). Nearly all of the gaps (17Mb of Ns) in the V2 assembly were filled, and our assembly contains ~25 Mb of new sequences. Because this improvement is so drastic and the old assembly had so many erroneous scaffolds, it’s difficult to assess the exact number of gaps that were filled.

37 gaps remained in the V4 assembly after BNG hybrid scaffolding. This includes 23kb of N’s with an average gap size of 621 bp. These gaps likely correspond to highly complex, repetitive regions that are difficult to assemble. These gaps may also include unanchored sequences that had no label sites in the BNG maps.

7. How many predicted genes in the new assembly can be supported by the RNA-seq data or can be supported by the predicted genes in V2? Maybe use a Venn diagram here? What's the reason(s) leading to those unshared genes?

Response: Out of the 28,588 total genes in the annotation, 27,491genes are supported by RNA-seq data. Also, out of the 1496 new genes, 1199 were supported with RNA-seq data. These newly identified genes, not shared in V2, either resided within the gaps in the V2 assembly or were collapsed tandem duplicates.

Minor:
1. In the manuscript, 'previous version' was mentioned several times. I think it is better to specify which version of Fragaria vesca genome assembly was used in the first appearance of the 'previous version'.

Response: We agree. The manuscript has been modified accordingly except instances referencing only new versions of the annotation.

2. I think it is better to use 'the second generation sequencing' to represent the short read sequencing rather than 'the next generation sequencing' (To my knowledge, PacBio sequencing also belongs to the next generation sequencing).

Response: We agree. The manuscript has been modified accordingly.

3. It is better to specify the version of all tools used in the manuscript rather than letting readers find them in the supplementary file.

Response: We have added these details to the manuscript for any tools with multiple

| | |
|---|---|
| | versions currently available.<br><br>4. It is good to use such as min read length, max read length, average read length and Std to show the stats of PacBio single molecules rather than giving the number of N50. I think N50 is mainly used to show the stats of contigs or scaffolds.<br><br>Response: The minimum read length was 3kb (reads shorter than this were filtered prior to assembly) and max read length was 72kb. We sequenced at total of 2,332,270 reads with an average read length of 8,295 bp. For distribution of reads see supplemental Figure 1. We have added these metrics to the manuscript. N50 subread length is commonly used to describe the length distribution of PacBio reads so we have left this in the text.<br><br>5. It will be good to specify which method was used to remove chloroplast and mitochondrial genomes? BLAST or others?<br><br>Response: We agree - this detail has been added to supplemental methods. BLAST was used to identify the organellar genomes.<br><br>***<br><br>Reviewer #2: 1. This manuscript provides a beautiful example of lots of existing short-read based genome sequences that need significant improvement so that more authentic biology can be revealed from studies and analysis based on the reference genome sequences.<br>2. The cost for 80X Pacbio sequence reads, and the optical maps generated by BioNanoGenomic systems could be a hurdle for lots of genome sequencing projects to gain all these kinds of datasets, therefore, new affordable technologies need to be in place in order to improve the quality for all genome sequencing projects.<br>3. Excited to see that more than 10% new sequences and genes were detected from new data and new assembly.<br>4. It is very interesting to see that how different genome assemblies affect the profiles of methylation and gene expression, and their effect on the biological explanation of the experiment result.<br><br>Response: We really appreciate your positive comments and feedback. In addition, we want to note that the cost of the entire project, both PacBio and BioNano, was under $15,000 USD total. The cost for these genome projects, especially relatively small plant genome projects of this size, are quite affordable now for even a single research programs or between collaborators. |
| **Additional Information:** | |
| **Question** | **Response** |
| Are you submitting this manuscript to a special series or article collection? | No |
| **Experimental design and statistics**<br><br>Full details of the experimental design and statistical methods used should be given in the Methods section, as detailed in our Minimum Standards Reporting Checklist. Information essential to interpreting the data presented should be made available in the figure legends.<br><br>Have you included all the information requested in your manuscript? | Yes |
| **Resources** | Yes |

| | |
|---|---|
| A description of all resources used, including antibodies, cell lines, animals and software tools, with enough information to allow them to be uniquely identified, should be included in the Methods section. Authors are strongly encouraged to cite Research Resource Identifiers (RRIDs) for antibodies, model organisms and tools, where possible.<br><br>Have you included the information requested as detailed in our Minimum Standards Reporting Checklist? | |
| **Availability of data and materials**<br><br>All datasets and code on which the conclusions of the paper rely must be either included in your submission or deposited in publicly available repositories (where available and ethically appropriate), referencing such data using a unique identifier in the references and in the "Availability of Data and Materials" section of your manuscript.<br><br>Have you have met the above requirement as detailed in our Minimum Standards Reporting Checklist? | Yes |

Manuscript

Click here to download Manuscript H4_Genome_Manuscript_Final3.pdf

**Title:** Single-molecule sequencing and optical mapping yields an improved genome of woodland strawberry (*Fragaria vesca*) with chromosome-scale contiguity

**Authors:** Patrick P. Edger[a,b,1,2], Robert VanBuren[a,1], Marivi Colle[a], Thomas J. Poorten[c], Ching Man Wai[a], Chad E. Niederhuth[d], Elizabeth I. Alger[a], Shujun Ou[a,b], Charlotte B. Acharya[c], Jie Wang[e], Pete Callow[a], Michael R. McKain[f], Jinghua Shi[g], Chad Collier[g], Zhiyong Xiong[h], Jeffrey P. Mower[i], Janet P. Slovin[j], Timo Hytönen[k], Ning Jiang[a,b], Kevin L. Childs[e,l], Steven J. Knapp[c,2]

a. Department of Horticulture, Michigan State University, East Lansing, MI
b. Ecology, Evolutionary Biology, and Behavior, Michigan State University, East Lansing, MI
c. Department of Plant Sciences, University of California - Davis, Davis, CA
d. Department of Genetics, University of Georgia, Athens, GA
e. Department of Plant Biology, Michigan State University, East Lansing, MI
f. Donald Danforth Plant Science Center, St. Louis, MO
g. Bionano Genomics, San Diego, CA
h. Potato Engineering & Technology Research Center, Inner Mongolia University, Hohhot, China
i. Center for Plant Science Innovation, University of Nebraska, Lincoln, NE
j. USDA/ARS, Genetic Improvement of Fruits and Vegetables Laboratory, Beltsville, MD
k. Department of Agricultural Sciences, Viikki Plant Science Centre, University of Helsinki, Helsinki, Finland
l. Center for Genomics Enabled Plant Science, Michigan State University, East Lansing, MI
1. PPE and RV contributed equally to this work
2. Author for correspondence: sjknapp@ucdavis.edu or edgerpat@msu.edu

**Abstract:** Although draft genomes are available for most agronomically important plant species, the majority are incomplete, highly fragmented, and often riddled with assembly and scaffolding errors. These assembly issues hinder advances in tool development for functional genomics and systems biology. Here we utilized a robust, cost-effective approach to produce high-quality reference genomes. We report a near-complete genome of diploid woodland strawberry (*Fragaria vesca*) using single-molecule real-time sequencing from Pacific Biosciences (PacBio). This assembly has a contig N50 length of ~7.9 Mb, representing a ~300 fold improvement of the previous version. The vast majority (>99.8%) of the assembly was anchored to seven pseudomolecules using two sets of optical maps from Bionano Genomics. We obtained ~24.96 million base pairs (Mb) of sequence not present in the previous version of the *F. vesca* genome and produced an improved annotation that includes 1,496 new genes. Comparative syntenic analyses uncovered numerous, large-scale scaffolding errors present in each chromosome in the previously published version of the *F. vesca* genome. Our results highlight the need to improve existing short-read based reference genomes. Furthermore, we demonstrate how genome quality impacts commonly used analyses for addressing both fundamental and applied biological questions.

Eukaryotic genomes, particularly plants, are notoriously difficult to assemble because of issues related to high repeat content, a history of gene and whole genome duplications, and regions of highly skewed nucleotide composition [1]. The short-reads (50-300 bp) generated by second generation sequencing technologies are often insufficient to resolve complex genomic features and regions. Short-reads are unable to span large repetitive regions resulting in sequence gaps and ambiguities in the assembly graph structures. Despite this known limitation, short-read sequencing platforms have been used for the majority of genome sequencing projects over the past decade resulting in a series of unfinished, fragmented draft genome assemblies [2]. For instance, the genome of woodland strawberry (*Fragaria vesca* 'Hawaii-4') was assembled using a mixture of different short read technologies and yielded 16,487 contigs in 3,263 scaffolds with an N50 length of ~27 kb [3] (version 1; V1). Dense linkage maps were later utilized to split multiple chimeric scaffolds and improve anchoring to the seven pseudomolecules [4]. However, the *F. vesca* (version 2; V2) genome remains incomplete with 6.99% gaps, missing megabase-sized regions, and scaffolding errors.

*Fragaria vesca* serves as an important model system for genetic studies for the Rosaceae community, due to its small stature, short generation time, a simple and efficient system for genetic transformation, and an increasing number of genetic resources [5–7]. With more than 2,500 described species, Rosaceae is one of the most speciose eudicot families and includes a breadth of important crops (e.g. almonds, apples, apricots, blackberries, cherries, peaches, pears, plums, raspberries, roses and strawberries) [8]. Furthermore, *F. vesca* is a valuable genetic resource because it is the putative diploid progenitor of the A subgenome of the cultivated octoploid strawberry (*F. x ananassa*) [9]. Strawberries are of major economic importance worldwide with 373,435 hectares planted and 8,114,373 metric tonnes of fruit produced in 2014 [10]. The *F. vesca* genome (V1 and V2) have been used to uncover underlying genetic factors regulating plant and fruit development, seasonal flowering, sex determination, metabolite diversity, and disease resistance [11–16]. A high-quality reference genome for *F. vesca* would further enable family-wide comparative studies and leverage the strengths offered by this model system for both fundamental and applied research.

We aimed to improve the *F. vesca* '*Hawaii-4*' reference genome using a long-read PacBio single-molecule real-time (SMRT) sequencing approach. We generated 2.3 million PacBio reads collectively spanning 19.4 Gb (80.8x coverage) with a subread N50 length of 9.2 kb and average length of 8.3 kb (Supplemental Figure 1; NCBI BioProject ID PRJNA383733). The minimum and maximum read lengths were 3kb and 72kb, respectively. The raw PacBio reads were error corrected and assembled using the Canu V1.4 [17] assembler followed by two rounds of polishing with Quiver V2.3.0 [18]. High coverage (~40x) Illumina data was aligned to the PacBio assembly and residual errors were corrected using Pilon V1.21 [19]. After removing the complete chloroplast and mitochondrial genomes, the final assembly spanned 219 Mb across 61 contigs with an N50 length of 7.9 Mb. Half of the assembly is contained in the largest 9 contigs, including five that exceed 10 Mb. The assembly graph is relatively simple with few ambiguities excluding a small cluster of five contigs corresponding to rRNA gene arrays from the nucleolar organizer region (Supplemental Figure 2). This represents a ~300 fold improvement in contiguity compared to the Illumina and 454 based *F. vesca* V1 assembly [3].

The PacBio based contigs were anchored into a chromosome-scale assembly using a two-enzyme BioNano genome map. Contigs were scaffolded first using the BsqQI map and this hybrid assembly was used as a reference for the BssSI map. The combined BioNano and PacBio assembly spans 220.8 Mb across 31 scaffolds with an N50 length of 36.1 Mb and 99.8% of the assembly captured in 9 scaffolds (Supplemental Table 1). Five of the seven *F. vesca* chromosomes are complete and two chromosomes were assembled into chromosome arms. The two pairs of chromosome arms were anchored using support from genetic maps [3]. The PacBio and BioNano assembly (hereon referred to as *F. vesca* V4) captures ~24.96 Mb of additional sequences with significant improvements in contiguity. *F. vesca* V4 has nine terminal telomere tracks with sequence and genome map support (**Figure 1**, Supplemental Figure 3), suggesting that the assembly is largely complete. Tandem arrays of centromeric repeats with monomeric lengths of 140, 143, and 147 bp were found in all seven chromosomes, consistent with V1 [3]. *Fragaria vesca* V4 contains three nucleolus organizer regions (NOR) at the beginning of Fvb1 and Fvb7 and at the end of Fvb5, consistent with previous cytological observations [20]. NOR rRNA arrays are complete on Fvb1 and Fvb5, but fragmented on Fvb7, based on sequence and genome map support. The 5S rRNA array is located 5 Mb upstream of the NOR on Fvb7 (Supplemental Figure 4). The *F. vesca* V4 assembly and annotation will be made publicly available on Genome Database for Rosaceae (https://www.rosaceae.org/), Phytozome (www.phytozome.net/) and CyVerse CoGe platform (https://genomevolution.org/).

A whole genome comparison of *F. vesca* V4 to V2 [4] uncovered numerous, large-scale scaffolding errors made in each of the chromosomes in the previous version (**Figure 2**). The overall quality of the *F. vesca* V4 assembly, compared to V2, is also supported by the distribution pattern of DNA methylation across chromosomes (Supplemental Figure 5). These types of errors considerably hinder various genomic analyses, including fine-mapping genes underlying traits [*21*] and identifying structural variants via comparative genomics. Here we demonstrate the superior quality of *F. vesca* V4 by making comparisons to a high-density linkage map of *Fragaria iinumae* [22], which is another putative diploid progenitor species of the cultivated octoploid strawberry. The total number of collinear markers against the *F. iinumae* genetic map increased by over 10% using *F. vesca* V4, compared to V2, and identified a distinctive chromosomal inversion between the two species near the pericentromeric region on chromosome 3 (Supplemental Figure 6, Supplemental Table 2, Table S1).

Although the quality of previous annotations of the *F. vesca* genome [3,23] is comparable to other annotations of short-read assemblies, they are, unavoidably, incomplete and fragmented resulting in errors in gene identification and gene number predictions [24]. Thus, despite the increasing volume of transcript and protein sequence information generated from various experimental studies, the task of improving genome annotation of such genomes remains a major challenge. Using the MAKER-P annotation pipeline [25], publicly available transcriptome data of *F. vesca,* and protein sequences from *Arabidopsis thaliana* and the UniprotKB database as evidence, we identified 28,588 gene models in *F. vesca* V4, of which 70% have a known Pfam domain. The mean length of the predicted genes is 1,475 bp (Supplemental Table 3). Repetitive elements were annotated, including long terminal repeat

retrotransposons (LTR-RTs) (e.g., *gypsy* and *copia*; **Figure 1**), non-LTR retrotransposons, and DNA transposons, using RepeatModeler [26], MITE_Hunter [27], and LTR_retriever [28]. Most repetitive elements are unassembled, incomplete or collapsed in short-read based reference genomes, which result in the underestimation of the repeat content of most eukaryotic genomes [29]. The improvement in genome quality of *F. vesca* V4 permitted the identification of additional LTR-RTs (Supplemental Table 4). Furthermore, an analysis of the insertion times of each LTR-RTs indicates that there were two major LTR-RT bursts; approximately 1.8 and 1.2 million years before present (Supplemental Figure 7). Organellar genomes from the plastid and mitochondrion were also annotated and verified for completeness (Supplemental Figures 8-9).

The Benchmarking Universal Single-Copy Orthologs (BUSCO V2 [30]) method was used to estimate the completeness of genome assembly and quality of gene annotation of *F. vesca* V4. The majority (95%) of the 1,440 core genes in the embryophyta dataset were identified in the annotation, which is supportive of a high-quality assembly and annotation similar to other high-quality grade genomes [31-33]. The overall quality of the annotation is further supported by the distribution of DNA methylation across the gene bodies (**Figure 3**). The *F. vesca* V4 annotation shows much sharper distribution patterns, especially in the CG context, and lower CHG and CHH (where H=A, T or C) methylation in the gene bodies. These patterns are expected for annotations that are more accurate and contain fewer mis-annotations (e.g., pseudogenes, transposons, etc). Additionally, *F. vesca* V4 contains 1,496 newly predicted gene models, with a mean length of 1,505 bp, that were not present in all previous versions of the annotation [3,23]. The vast majority of these new genes (1,463 total) are expressed in different fruit tissues and developmental stages (**Figure 4;** Table S2). Thus, previous expression studies may have missed key genes controlling fruit development and maturation in *F. vesca* [34,35]. Of the new genes in *F. vesca* V4, 810 genes did not show similarity at the protein level (query length < 30%, E= $10^{-10}$) to any paralogs in the V2 genome but exhibit unique expression patterns (**Figure 4**). We also identified significantly more tandemly duplicated genes and larger tandem arrays in *F. vesca* V4 (Supplemental Figure 10). Long-read single molecule sequencing approaches have been shown to better resolve tandemly repeated copies [36–38]. The identification of tandemly duplicated genes is important since such genes are highly enriched for both abiotic and biotic stress related functions [39]. For example, many important plant defense genes, including nucleotide-binding site leucine-rich repeat (*NBS-LRR*) [40] and cytochrome p450s (*CYPs*) [41], are tandemly duplicated and exhibit high levels of copy number variation within a species.

Here we present one of the most complete and contiguous plant genomes assembled to date. The average published plant genome is highly fragmented with a contig N50 length of roughly 50kb [2], compared to ~7.9Mb for *F. vesca* V4. The *F. vesca* V4 genome has the third best contig N50 of any angiosperm sequenced to date, after only *Arabidopsis thaliana* [42] and *rice* (*Oryza sativa*) [43]. It is important to note that the total cost for a PacBio sequenced and BioNano Genomics genome is a very small fraction of the cost compared to these Sanger era genomes [31]. Our genomic analyses, which included direct comparisons to previously published versions of the same genotype [3,4,23], highlight the need to improve existing short-read based reference genomes. The approach used here, combining long-read sequencing and

optical maps, correct mis-assembly and scaffolding errors commonly found in short-read based genomes, which dramatically impact the results in genetic mapping (Supplemental Figure 6), methylation (**Figure 3**), and gene expression studies (**Figure 4**).

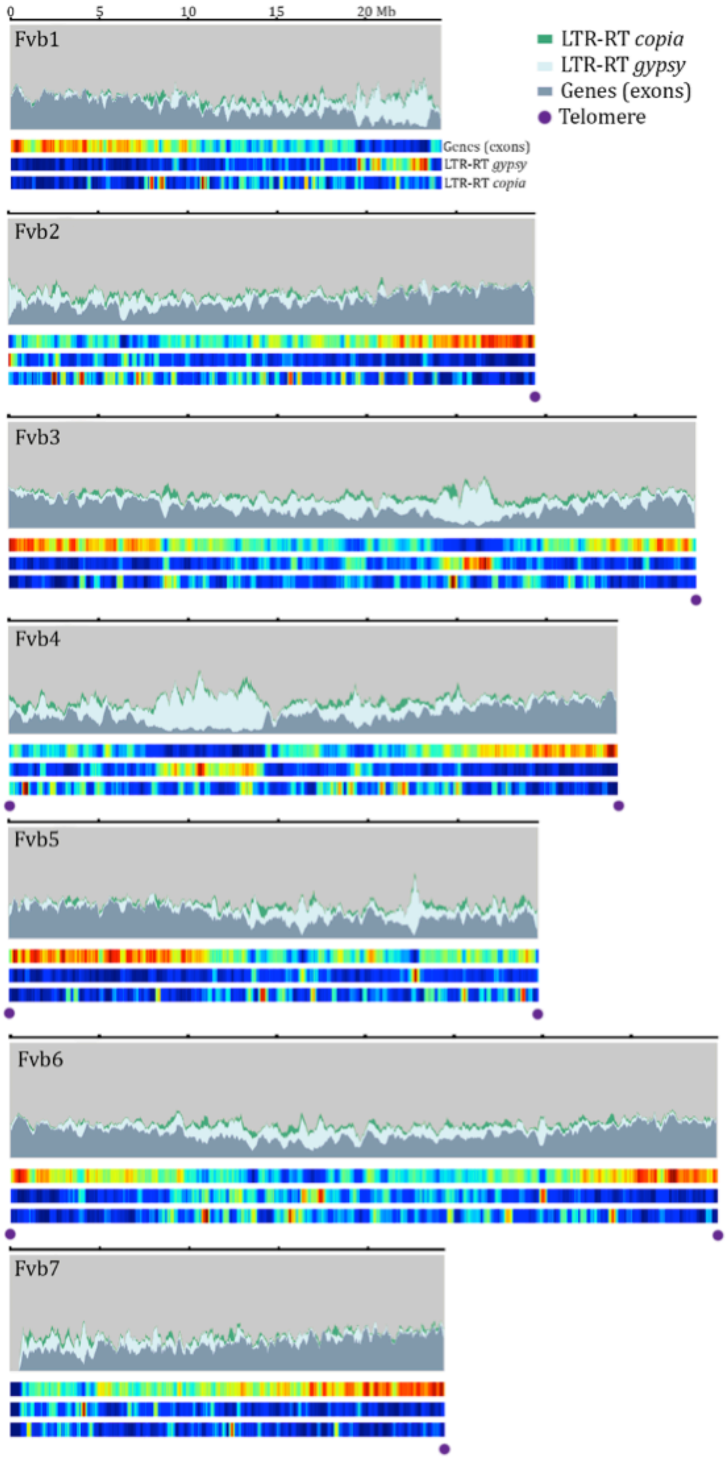


**Figure 1. Chromosome landscapes of the *F. vesca* V4 genome**

The distribution of genes and long terminal repeat retrotransposons (LTR-RTs) are plotted for each of the seven chromosomes. Heatmaps reflect the distribution of elements with blue indicating the lowest abundance and red signifying high abundance. Plots were generated with sliding window of 50kb with 10kb shift across each chromosome. Terminal telomeric repeat arrays are denoted in purple.

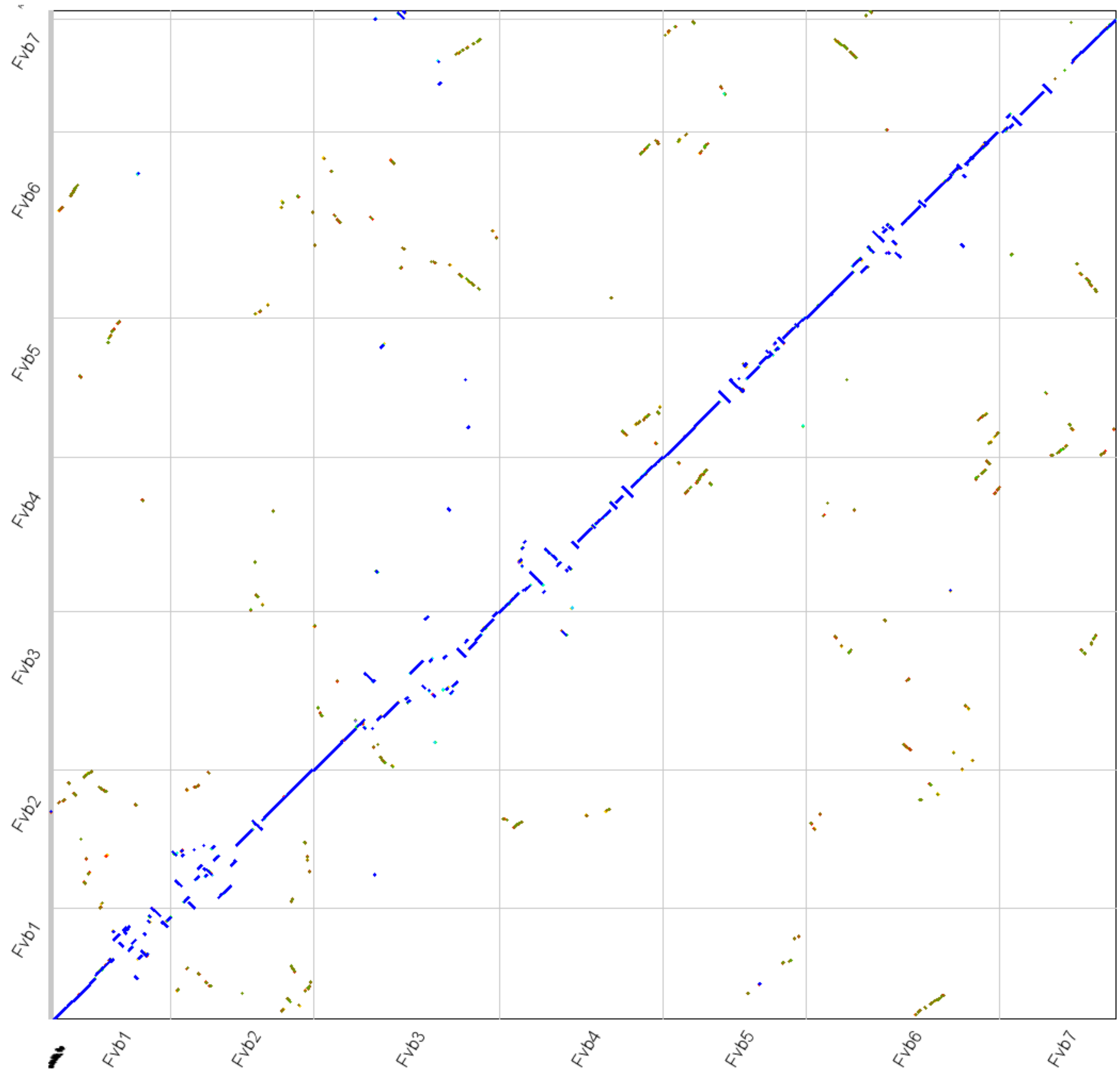


**Figure 2. Macrosyntenic comparison of the V2 and V4 *F. vesca* assemblies**
Syntenic gene pairs between V4 (x-axis) and V2 (y-axis) of *F. vesca* were identified by DAGChainer[44], sorted by chromosome (Fvb1-7), and colored based on their synonymous substitution rate as calculated by CodeML[45] using SynMap within CoGe[46]. Syntenic 'orthologous' regions are colored in blue and duplicated genes retained from a whole genome triplication event (At-gamma[47]) in other colors. Regions that were misassembled and incorrectly scaffolded in *F. vesca* V2 are identified by negatively sloped and repositioned lines.

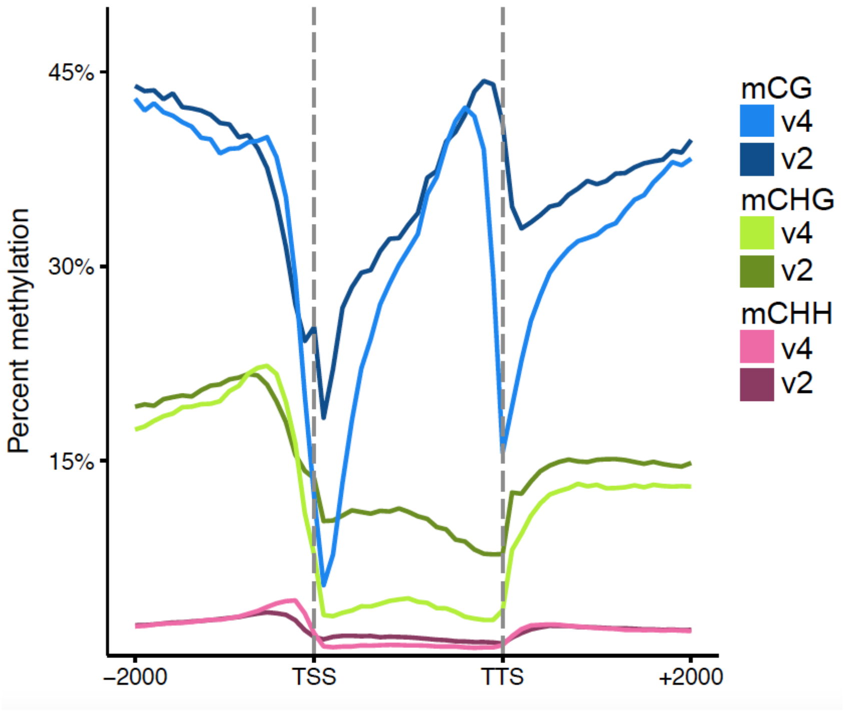


**Figure 3**: **Distribution of gene body methylation in the V2 and V4 *F. vesca* assemblies.** This plot shows the average DNA methylation patterns (CG = Blue, CHG = Green, CHH = Red; H=A, T or C) across all genes in the V2 (darker colors) and V4 (lighter colors) assemblies. The X-axis shows the transcription start sites (TSS, left dashed line) and the transcription termination sites (TTS, right dashed line), plus +/- 2000 bp from each gene.

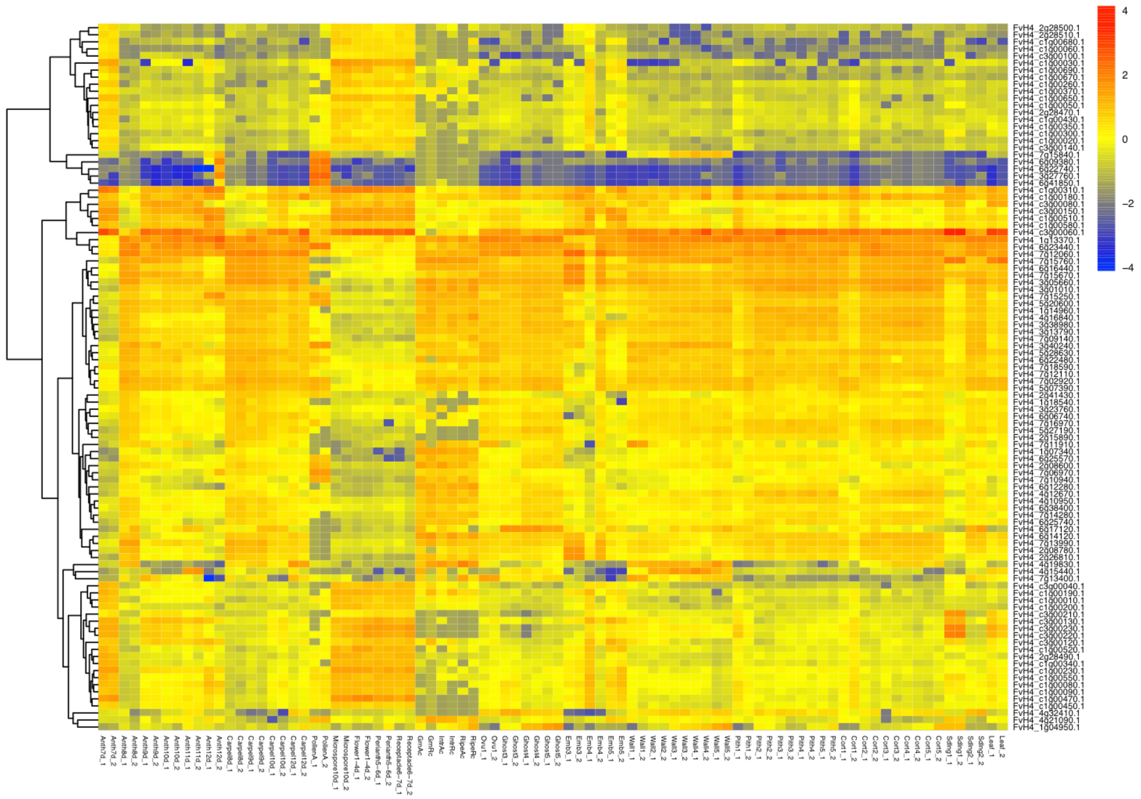


**Figure 4**: **Expression patterns of newly annotated genes across diverse tissue types**
Heatmap consists of a random subset of 100 genes from the unique 810 newly identified genes in the *F. vesca* V4 assembly, across 22 tissue types at different developmental stages. Two biological replicates were sequenced per tissue with the exception of six with only one biological replicate each (Table S2). Blue indicates the lowest expression and red signifies the highest expression abundance. Gene expression level was calculated based on RPKM (Reads Per Kilobase of transcript per Million mapped reads) and visualized through heatmap analysis using variance stabilized transformed values on a log2 scale.

## References


1. Schatz, M. C., Witkowski, J. & McCombie, W. R. Current challenges in *de novo* plant genome sequencing and assembly. *Genome Biol.* **13,** (2012).
2. Michael, T. P. & VanBuren, R. Progress, challenges and the future of crop genomes. *Curr. Opin. Plant Biol.* **24,** 71–81 (2015).
3. Shulaev, V. *et al.* The genome of woodland strawberry (*Fragaria vesca*). *Nat. Genet.* **43,** 109–116 (2011).
4. Tennessen, J. A., Govindarajulu, R., Liston, A. & Ashman, T.-L. Targeted Sequence Capture Provides Insight into Genome Structure and Genetics of Male Sterility in a Gynodioecious Diploid Strawberry, *Fragaria vesca* ssp *bracteata* (Rosaceae). *G3* **3,** 1341–1351 (2013).
5. Folta, K. M. & Davis, T. M. Strawberry genes and genomics. *CRC Crit. Rev. Plant Sci.* **25,** 399–415 (2006).
6. Liston, A., Cronn, R. & Ashman, T.-L. *Fragaria*: A genus with deep historical roots and ripe for evolutionary and ecological insights. *Am. J. Bot.* **101,** 1686–1699 (2014).
7. Slovin, J. P. & Michael, T. P. Strawberry Part 3-structural and functional genomics. *Genetics, genomics and breeding of berries* 240–308 (2011).
8. Shulaev, V. *et al.* Multiple models for Rosaceae genomics. *Plant Physiol.* **147,** 985–1003 (2008).
9. Senanayake, Y. D. & Bringhurst, R. S. Origin of *Fragaria* Polyploids. I. Cytological Analysis. *Am. J. Bot.* **54,** 221 (1967).
10. Faostat, F. Agriculture Organization of the United Nations Statistics Division (2014). Production Available in: http://faostat3.fao.org/browse/Q/QC/S [Review date: April 2015] (2016).
11. Ashman, T.-L. *et al.* Multilocus Sex Determination Revealed in Two Populations of Gynodioecious Wild Strawberry, *Fragaria vesca* subsp. *bracteata*. *G3* **5,** 2759–2773 (2015).
12. Koskela, E. *et al*. Mutation in *TERMINAL FLOWER1* reverses the photoperiodic requirement for flowering in the wild strawberry, *Fragaria vesca*. *Plant Phys.* **159**, 1043–1054 (2012).
13. Naithani, S., Partipilo, C. M., Raja, R., Elser, J. L. & Jaiswal, P. FragariaCyc: A Metabolic Pathway Database for Woodland Strawberry *Fragaria vesca*. *Front. Plant Sci.* **7,** 242 (2016).
14. Tennessen, J. A., Govindarajulu, R., Liston, A. & Ashman, T.-L. Homomorphic ZW chromosomes in a wild strawberry show distinctive recombination heterogeneity but a small sex-determining region. *New Phytol.* **211,** 1412–1423 (2016).
15. Wei, W. *et al.* The WRKY transcription factors in the diploid woodland strawberry *Fragaria vesca*: Identification and expression analysis under biotic and abiotic stresses. *Plant Physiol. Biochem.* **105,** 129–144 (2016).
16. Chen, X.-R., Brurberg, M. B., Elameen, A., Klemsdal, S. S. & Martinussen, I. Expression of resistance gene analogs in woodland strawberry (*Fragaria vesca*) during infection with *Phytophthora cactorum*. *Mol. Genet. Genomics* **291,** 1967–1978 (2016).
17. Koren, S. *et al.* Canu: scalable and accurate long-read assembly via adaptive k-mer weighting and repeat separation. *Genome Res.* (2017). doi:10.1101/gr.215087.116

18. Chin, C.-S. *et al.* Nonhybrid, finished microbial genome assemblies from long-read SMRT sequencing data. *Nat. Methods* **10,** 563 (2013).
19. Walker, B. J. *et al.* Pilon: An Integrated Tool for Comprehensive Microbial Variant Detection and Genome Assembly Improvement. *PLoS One* **9,** (2014).
20. Liu, B. & Davis, T. M. Conservation and loss of ribosomal RNA gene sites in diploid and polyploid *Fragaria* (Rosaceae). *BMC Plant Biol.* **11,** (2011).
21. Samad, S. *et al.* Additive QTLs on three chromosomes control flowering time in woodland strawberry (*Fragaria vesca* L.). *Hort. Res.,* in press (2017).
22. Mahoney, L. L. *et al.* A High-Density Linkage Map of the Ancestral Diploid Strawberry, *Fragaria iinumae*, Constructed with Single Nucleotide Polymorphism Markers from the IStraw90 Array and Genotyping by Sequencing. *Plant Genome* **9,** (2016).
23. Darwish, O., Shahan, R., Liu, Z., Slovin, J. P. & Alkharouf, N. W. Re-annotation of the woodland strawberry (*Fragaria vesca*) genome. *BMC Genomics* **16,** (2015).
24. Yandell, M. & Ence, D. A beginner's guide to eukaryotic genome annotation. *Nat. Rev. Genet.* **13,** 329–342 (2012).
25. Campbell, M. S. *et al.* MAKER-P: A Tool Kit for the Rapid Creation, Management, and Quality Control of Plant Genome Annotations. *Plant Physiol.* **164,** 513–524 (2014).
26. Smit, A. & Hubley, R. RepeatModeler Open-1.0. *Repeat Masker Website* (2010).
27. Han, Y. & Wessler, S. R. MITE-Hunter: a program for discovering miniature inverted-repeat transposable elements from genomic sequences. *Nucleic Acids Res.* **38,** e199 (2010).
28. Ou, S. & Jiang, N. LTR_retriever: a highly accurate and sensitive program for identification of LTR retrotransposons. bioRvix (2017)
29. de Koning, A. P. J., Gu, W., Castoe, T. A., Batzer, M. A. & Pollock, D. D. Repetitive Elements May Comprise Over Two-Thirds of the Human Genome. *PLoS Genet.* **7,** (2011).
30. Simão, F. A., Waterhouse, R. M., Ioannidis, P., Kriventseva, E. V. & Zdobnov, E. M. BUSCO: assessing genome assembly and annotation completeness with single-copy orthologs. *Bioinformatics* **31,** 3210–3212 (2015).
31. VanBuren, R. *et al.* Single-molecule sequencing of the desiccation-tolerant grass *Oropetium thomaeum*. *Nature* **527,** 508–U209 (2015).
32. Jarvis, D. E. *et al.* The genome of *Chenopodium quinoa*. *Nature* **542,** 307 (2017).
33. Bickhart, D. M. *et al.* Single-molecule sequencing and chromatin conformation capture enable *de novo* reference assembly of the domestic goat genome. *Nat. Genet.* **49,** 643 (2017).
34. Hollender, C. A., Geretz, A. C., Slovin, J. P. & Liu, Z. Flower and early fruit development in a diploid strawberry, *Fragaria vesca*. *Planta* **235,** 1123–1139 (2012).
35. Kang, C. *et al.* Genome-Scale Transcriptomic Insights into Early-Stage Fruit Development in Woodland Strawberry *Fragaria vesca*. *Plant Cell* **25,** 1960–1978 (2013).
36. Krsticevic, F. J., Schrago, C. G. & Carvalho, A. B. Long-Read Single Molecule Sequencing to Resolve Tandem Gene Copies: The *Mst77Y* Region on the *Drosophila melanogaster* Y Chromosome. *G3* **5,** 1145–1150 (2015).
37. Torresen, O. K. *et al.* An improved genome assembly uncovers prolific tandem repeats in Atlantic cod. *BMC Genomics* **18,** (2017).
38. Oren, M. *et al.* Short tandem repeats, segmental duplications, gene deletion, and genomic instability in a rapidly diversified immune gene family. *BMC Genomics* **17,** (2016).

39. Edger, P. P. & Pires, J. C. Gene and genome duplications: the impact of dosage-sensitivity on the fate of nuclear genes. *Chromosome Res.* **17,** 699–717 (2009).
40. McHale, L., Tan, X. P., Koehl, P. & Michelmore, R. W. Plant NBS-LRR proteins: adaptable guards. *Genome Biol.* **7,** (2006).
41. Hofberger, J. A., Lyons, E., Edger, P. P., Pires, J. C. & Schranz, M. E. Whole Genome and Tandem Duplicate Retention Facilitated Glucosinolate Pathway Diversification in the Mustard Family. *Genome Biol. Evol.* **5,** 2155–2173 (2013).
42. Kaul, S. *et al.* Analysis of the genome sequence of the flowering plant *Arabidopsis thaliana*. *Nature* **408,** 796–815 (2000).
43. Matsumoto, T. *et al.* The map-based sequence of the rice genome. *Nature* **436,** 793–800 (2005).
44. Haas, B. J., Delcher, A. L., Wortman, J. R. & Salzberg, S. L. DAGchainer: a tool for mining segmental genome duplications and synteny. *Bioinformatics* **20,** 3643–3646 (2004).
45. Yang, Z. PAML: a program package for phylogenetic analysis by maximum likelihood. *Comput. Appl. Biosci.* **13,** 555–556 (1997).
46. Lyons, E., Pedersen, B., Kane, J. & Freeling, M. The Value of Nonmodel Genomes and an Example Using SynMap Within CoGe to Dissect the Hexaploidy that Predates the Rosids. *Trop. Plant Biol.* **1,** 181–190 (2008).
47. Bowers, J. E., Chapman, B. A., Rong, J. K. & Paterson, A. H. Unravelling angiosperm genome evolution by phylogenetic analysis of chromosomal duplication events. *Nature* **422,** 433–438 (2003).

**Author Contributions**: P.P.E., R.V. and S.J.K. designed research; P.P.E., R.V., M.C., T.J.P., C.M.W., C.E.N., E.A., S.O., C.B.A., J.W., P.C., M.R.M., J.S., C.C., Z.X., J.P.M., J.P.S., T.H., N.J., K.L.C., and S.J.K. performed research and/or analyzed data; and P.P.E., R.V., M.C., E.A. and S.J.K wrote the paper. All Authors reviewed the manuscript.

**Competing Interests**: The authors declare that they have no competing interests.

Click here to access/download
**Supplementary Material**
Supplement-H4GenomePaper_Final2.pdf

Table S1

Click here to access/download

**Supplementary Material**

H4_TableS1.xlsx

Table S2

Click here to access/download
**Supplementary Material**
H4_TableS2.xlsx
