# Supplementary material for: Single-molecule sequencing and optical mapping yields an improved genome of woodland strawberry (Fragaria vesca) with chromosome-scale contiguity
Source: Gigascience. 2017 Dec 13;7(2):gix124. doi: 10.1093/gigascience/gix124 (PMC5801600; doi:10.1093/gigascience/gix124)
Supplement: Supplemental data [file gix124_supp.zip › Supplement-H4GenomePaper_Final3.docx]

**Title:** Single-molecule sequencing and optical mapping yields an improved genome of woodland strawberry (*Fragaria vesca*) with chromosome-scale contiguity

**Authors:** Patrick P. Edger^a,b,1,2^**,** Robert VanBuren^a,1^, Marivi Colle^a^, Thomas J. Poorten^c^, Ching Man Wai^a^, Chad E. Niederhuth^d^, Elizabeth Alger^a^, Shujun Ou^a,b^, Charlotte B. Acharya^c^, Jie Wang^e^, Pete Callow^a^, Michael R. McKain^f^, Jinghua Shi^g^, Chad Collier^g^, Zhiyong Xiong^h^, Jeffrey P. Mower^i^, Janet P. Slovin^j^, Timo Hytönen^k^, Ning Jiang^a,b^, Kevin L. Childs^e,l^, Steven J. Knapp^c,2^

a. Department of Horticulture, Michigan State University, East Lansing, MI

b. Ecology, Evolutionary Biology, and Behavior, Michigan State University, East Lansing, MI

c. Department of Plant Sciences, University of California - Davis, Davis, CA

d. Department of Genetics, University of Georgia, Athens, GA

e. Department of Plant Biology, Michigan State University, East Lansing, MI

f. Donald Danforth Plant Science Center, St. Louis, MO

g. Bionano Genomics, San Diego, CA

h. Potato Engineering & Technology Research Center, Inner Mongolia University, Hohhot, China

i. Center for Plant Science Innovation, University of Nebraska, Lincoln, NE

j. USDA/ARS, Genetic Improvement of Fruits and Vegetables Laboratory, Beltsville, MD

k. Department of Agricultural Sciences, Viikki Plant Science Centre, University of Helsinki, Helsinki, Finland

l. Center for Genomics Enabled Plant Science, Michigan State University, East Lansing, MI

1. PPE and RV contributed equally to this work

2. Author for correspondence: [sjknapp@ucdavis.edu](mailto:sjknapp@ucdavis.edu) or [edgerpat@msu.edu](mailto:edgerpat@msu.edu)

**Supplemental Materials**

Supplemental Text S1: Genome Assembly

Supplemental Text S2: Methylation Analyses

Supplemental Text S3: *F. vesca* V4 comparisons to *Fragaria iinumae* linkage map

Supplemental Text S4: Genome Annotation

Supplemental Text S5: Organellar Genome Annotation

Supplemental Text S6: Gene-expression analysis

Supplemental Text S7: Synteny and comparative genomics

**Supplemental Text S1: Genome Assembly**

Raw PacBio reads, available to download from NCBI (BioProject ID:PRJNA383733), were error corrected and assembled using the Canu (V1.4)[^1^](https://paperpile.com/c/nMZe3a/SJEU) assembler with the following parameters: minReadLength=2000, GenomeSize=240Mb, minOverlapLength=1000. Other parameters were left as default. Contigs were polished using a reiterative approach with Quiver (V2.3.0)[^2^](https://paperpile.com/c/nMZe3a/od47) with a minimum sub-read length = 3000bp, minimum polymerase read quality= 0.8, maximum divergence percentage = 30 and minimum anchor size = 15. The polished contigs had ~99.9% sequence homology to the *F. vesca* V1 genome assembly, indicating the presence of residual errors. Contigs were polished using Illumina data from the short read archive (SRA; SRR3089069 and BioSample SAMN00120060) and Pilon (V1.21)[^3^](https://paperpile.com/c/nMZe3a/UePc). Quality trimmed Illumina reads were aligned to the Quiver polished contigs using bowtie2 (V2.3.0)[^4^](https://paperpile.com/c/nMZe3a/xZi7) with default parameters. The read mapping rate was ~98%, further supporting the completeness of our assembly. Reads were locally realigned around insertions/deletions (indels) using the IndelRealigner from the genome analysis tool kit (GATK; V3.7)[^5^](https://paperpile.com/c/nMZe3a/I2Px). Pilon parameters were as follows: --flank 7, --K 51, --mindepth 20. Pilon corrected a total of 24,325 indels spanning 45,345bp and 3,245 SNPs. Contigs corresponding to the mitochondria and chloroplast genomes were removed from the assembly prior to scaffolding. These organellar genomes were identified using nucleotide BLAST.


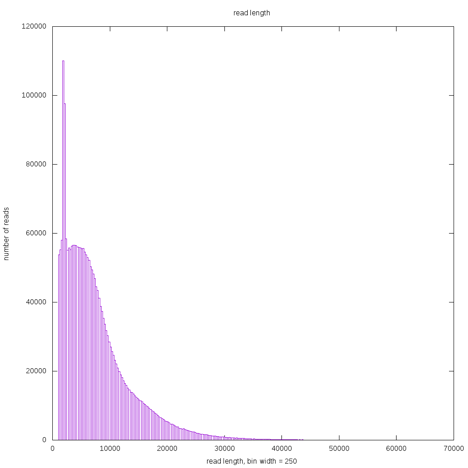


**Supplemental Figure 1** Histogram of PacBio read length distribution.


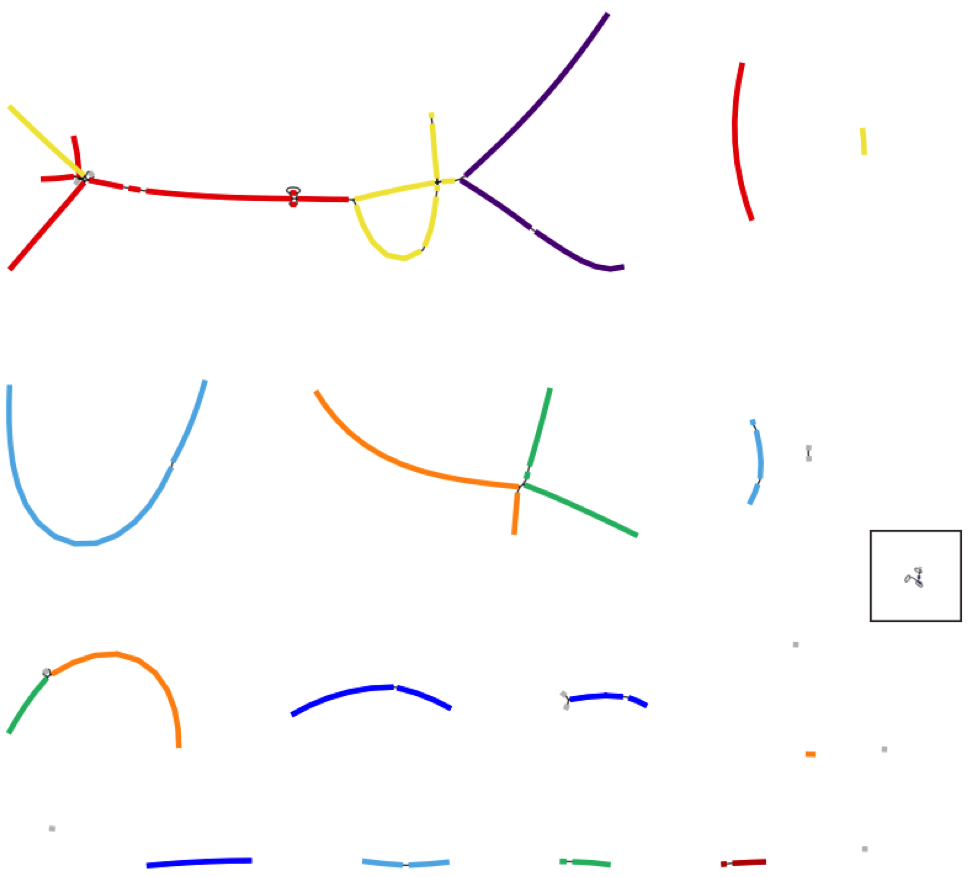


**Supplemental Figure 2** *de novo* assembly graph of the *F. vesca* V4 genome. Each line (node) represents a contig with connections (edges) representing ambiguities in the graph structure. Contigs are color coded by chromosome as follows: Fvb1 blue, Fvb2 purple, Fvb3 light blue, Fvb4 orange, Fvb 5 yellow, Fvb6 red, Fvb 7 green. Grey contigs are unanchored. Contigs corresponding to tandem arrays of ribosomal RNA genes in the nucleolus organize region (NOR) are highlighted (gray box).


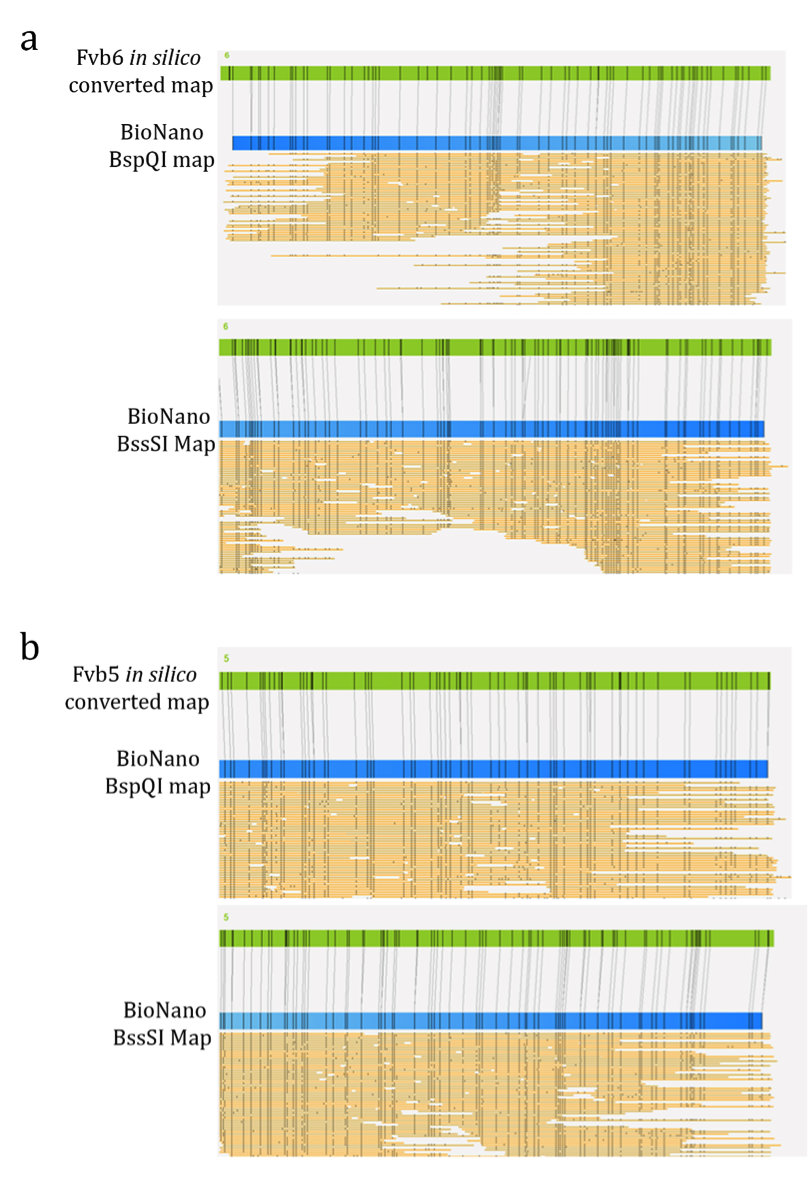


**Supplemental Figure 3** Bionano genome maps terminalize at the end of chromosomes. A majority of the tagged DNA molecules terminalize at the physical end of the chromosome sequences, suggesting chromosomes are complete.


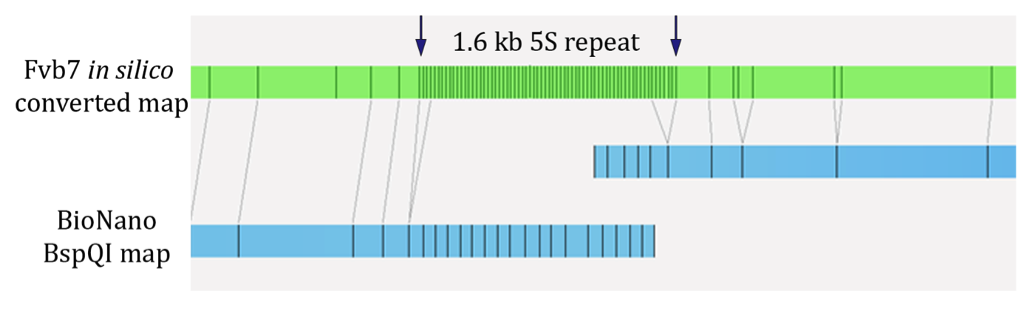


**Supplemental Figure 4** Sequence and genome map evidence of the 5S rRNA array. The 1.6kb array of 5S rRNA is denoted by blue arrows.

**Supplemental Table 1 Summary statistics of the raw BioNano genome maps**


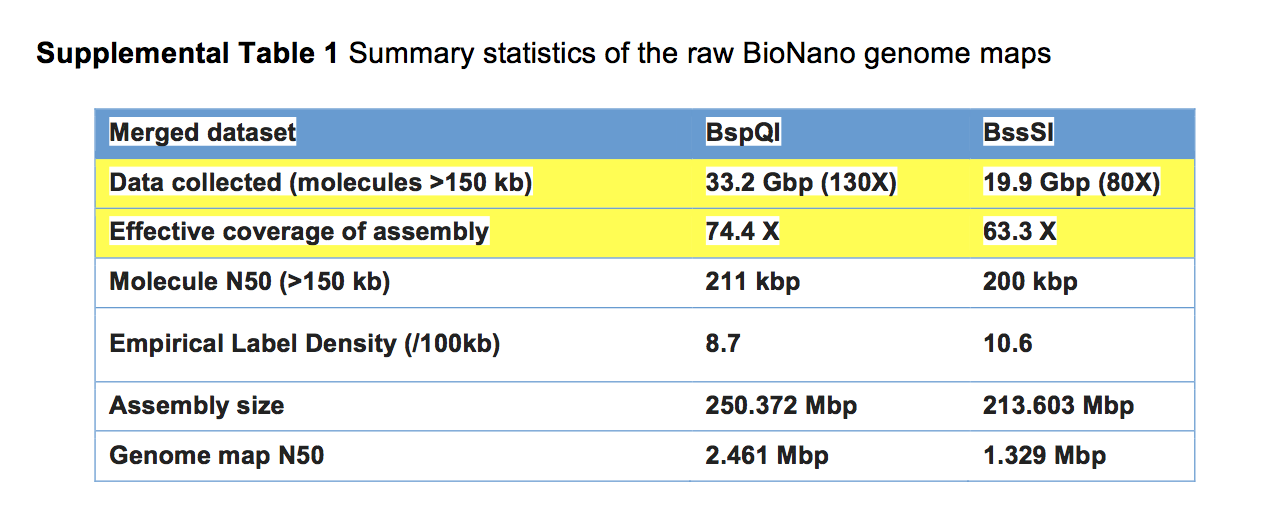


**Supplemental Table 2** Summary statistics of the combined BioNano genome map.


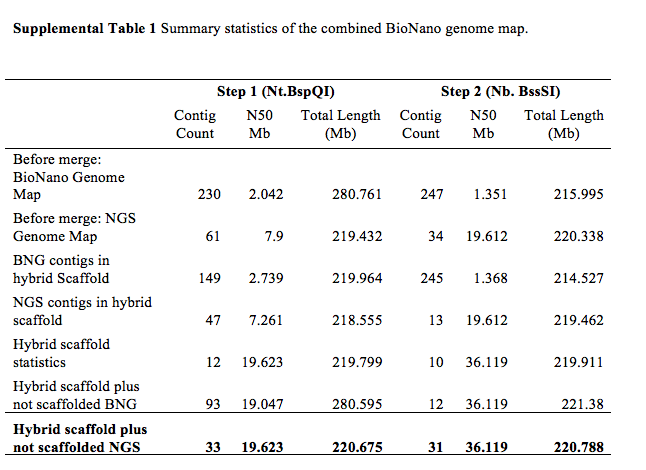


**Supplemental Text S2: Methylation Analyses**

Whole genome bisulfite sequencing from F. vesca Hawaii-4 (NCBI SRA: SRR3286267)[^6^](https://paperpile.com/c/nMZe3a/0VFF) was mapped to the v2 and v4 genomes and methylated sites called using previously described methods[^6,7^](https://paperpile.com/c/nMZe3a/7tde+0VFF). Custom python scripts were used to plot DNA methylation across chromosomes and gene bodies (available at: https://github.com/chadn737/Fvesca-v4- genome-paper). Briefly, for chromosome plots (Supplemental Figure 5), the weighted methylation level[^8^](https://paperpile.com/c/nMZe3a/xARl) was calculated for 100 Kbs sliding windows with a 50 Kbs step size. For gene metaplots, the gene body and 2000 bps upstream and downstream were each divided into 20 windows and methylation data mapped to the windows (**Figure 3**). For the gene bodies, only methylation within coding sequences was used as methylation of transposons located in UTR and intronic sequences can inflate methylation levels. These were then plotted in R using ggplot2[^9^](https://paperpile.com/c/nMZe3a/hTrk).


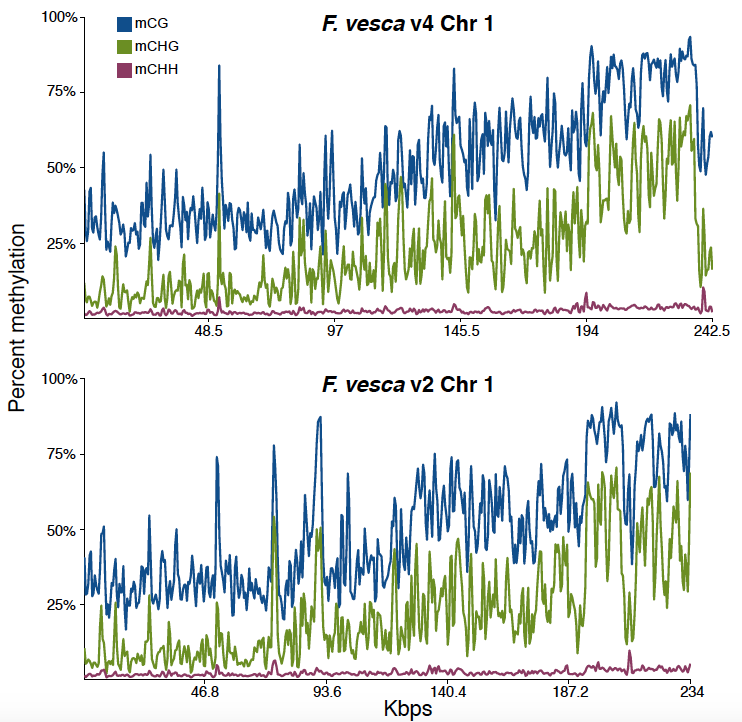


**Supplemental Figure 5** Comparison of DNA Methylation across Chromosome 1 between *Fragaria vesca* V2 & V4. The plot also shows chromosome size differences between V2 & V4 .

**Supplemental Text S3: *F. vesca* V4 comparisons to *Fragaria iinumae* linkage map**

The *F. iinumae* genetic map markers (4,173 total)[^10^](https://paperpile.com/c/nMZe3a/Ma3n) were mapped to *F. vesca* “Hawaii 4” version 4 genome using blastn. Hits with bit score lower than 60 and e-value smaller than e^-5^ were discarded. Markers with only one highest hit are used in synteny and colinearity comparison. The chromosomal position mapped, synteny and collinearity results are detailed in Table S1. The colinearity of chromosome 3 between *F. iinumae*, *F. vesca* V2 genome (ftp://ftp.bioinfo.wsu.edu/species/Fragaria_vesca/Fvesca-genome.v2.0.a1), and *F. vesca* V4 genome were plotted by strudel version 1.15.08.25 for visualization[^11^](https://paperpile.com/c/nMZe3a/pHO2). Among the 4,173 markers from *F. iinumae* genetic maps, 4,033 were unique mapped to *F. vesca* version 4 genome, 71 were mapped to more than one region with equal bit score, and 69 were not mapped to any region of *F. vesca* version 4 genome. Out of these 4,033 unique mapped markers, 3,976 syntenic and 3,918 colinear markers were observed (Supplemental Table 2).

Compare to alignment against *F. vesca* version 2 genome, the number of colinear markers between *F. iinumae* and *F. vesca* was increased from 3,490 to 3,918. By plotting the syntenic markers along chromosome 3, two major discrepancies between version 2.0 and version 4 alignments were observed. Firstly, genetic markers located between 55.365 - 57.785 cM of *F. iinumae* was colinear to 8.2 – 12.2 Mb of *F. vesca* genome version 4. However, these markers were aligned into two separate regions on *F. vesca* genome version 2.0 (8.2 -10.4 Mb and 18.4-20.2 Mb; Supplemental Figure 6). Secondly, an clear chromosomal inversion between *F. iinumae* and *F. vesca* genome version 4 was detected at 59.248 – 60.219 cM, which is equivalent to 12.3 – 30.9 Mb of *F. vesca* chromosome 3. This inversion event is less distinctive in *F. vesca* version 2 due to the genome misassembly located at 8.2 -10.4 and 18.4 – 20.2 Mb.

**Table S1 Chromosomal position of *F. iinumae* genetic map markers on *F. vesca* v4**

The marker sequences of *F. iinumae* genetic map were mapped to *F. vesca* v4 genome using blastn. Only uniquely mapped markers are used for syntenic and colinearity analysis. Syntenic and collinear markers are indicated as “x”. For markers with more than one mapped region with same hit scores are labeled as “multi-mapped markers” (MM). For markers with no mapped hit or bit scores lower than 60 are labeled as “unmapped markers” (unmapped). Markers that are mapped to chromosome non-syntenically are labeled as “others” (O). Information from column A to E is adopted from Supplementary Table 3 of Mahoney et al. (2016)[^10^](https://paperpile.com/c/nMZe3a/Ma3n).

**Supplemental Table 2: Summary of syntenic and collinear markers between *F. iinumae* genetic map, *F. vesca* V2 and V4 genome**

| **Fii LG number** | **Fii genetic map** | **Number of Markers** | | | | | |
| --- | --- | --- | --- | --- | --- | --- | --- |
|  |  | **Fvb** | | | **FvH4 v4** | | |
|  |  | **Syntenic** | **Colinear** | **Non-colinear** | **Syntenic** | **Colinear** | **Non-colinear** |
| **1** | **472** | **426** | **416** | **10** | **449** | **448** | **1** |
| **2** | **595** | **537** | **453** | **84** | **567** | **563** | **4** |
| **3** | **657** | **593** | **512** | **81** | **626** | **587** | **39** |
| **4** | **496** | **439** | **414** | **25** | **465** | **456** | **9** |
| **5** | **576** | **535** | **462** | **73** | **551** | **550** | **1** |
| **6** | **938** | **865** | **821** | **44** | **894** | **891** | **3** |
| **7** | **439** | **415** | **412** | **3** | **424** | **423** | **1** |
| **Total** | **4173** | **3810** | **3490** | **320** | **3976** | **3918** | **58** |

Information of Fii LG number, Fii genetic map and Fvb column is adopted from Mahoney et al. (2016)[^10^](https://paperpile.com/c/nMZe3a/Ma3n). Fvb refers to *F. vesca* version 2.0 genome obtained from Genome Database for Rosaceae (https://www.rosaceae.org/species/fragaria_vesca/genome_v2.0.a1).


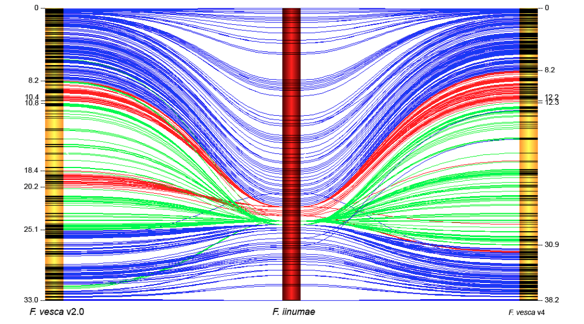


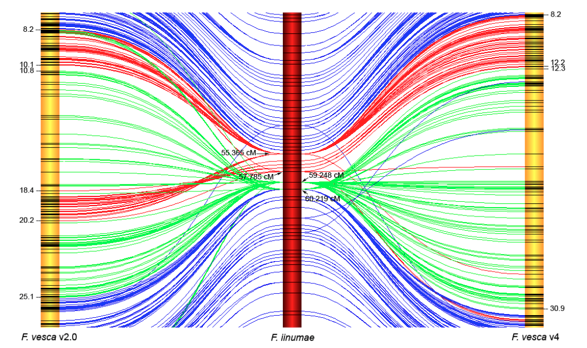


**Supplemental Figure 6: Colinearity between *F. vesca* chromosome 3 and *F. iinumae* linkage group 3** The overall colinearity (a) and enlarged view of 8 – 33 Mb (b) between *F. iinumae* linkage group 3 (middle), *F. vesca* chromosome 3 from version 2 assembly (left), and *F. vesca* chromosome 3 from version 4 assembly (right).

**Supplemental Text S4: Genome Annotation**

Genome annotation was performed using MAKER-P annotation pipeline[^12^](https://paperpile.com/c/nMZe3a/gvZB). Evidence sequences used for the annotation included protein sequences downloaded from *Arabidopsis thaliana* (Araport11)[^13^](https://paperpile.com/c/nMZe3a/bPvX) and UniprotKB plant databases[^14^](https://paperpile.com/c/nMZe3a/Z4ZF), *F. vesca* expressed sequence tags (EST) from NCBI, and mRNA-seq data, assembled by Trinity[^15^](https://paperpile.com/c/nMZe3a/C41p) and StringTie[^16^](https://paperpile.com/c/nMZe3a/X8vC) using available short reads from *F. vesca* plant tissues in NCBI Short Read Archive (SRA)(**Table S2**). Low complexity regions in the genome were soft-masked while complex repetitive regions were hard-masked using Repeatmasker. Custom Long Terminal Repeats (LTR) library (see LTR annotation section), and Repbase[^17^](https://paperpile.com/c/nMZe3a/I5uH) and MAKER’s repeat libraries[^18^](https://paperpile.com/c/nMZe3a/hgnu) were used for masking. *Ab initio* gene prediction was done using gene predictors, SNAP[^19^](https://paperpile.com/c/nMZe3a/SJOF) and Augustus[^20^](https://paperpile.com/c/nMZe3a/lzML), which were iteratively trained for *F. vesca*. The resulting MAKER Max gene set was filtered to select gene models with Pfam domain and annotation edit distance (AED) < 1.0. The filtered gene set (MAKER standard) was further scanned for transposase coding regions. The amino acid sequence of predicted genes was searched (BLASTP, E= 10-5) against a transposase database from a previous study[^12^](https://paperpile.com/c/nMZe3a/gvZB). The alignment between the genes and the transposases was further filtered for those caused by the presence of sequences with low complexity. Briefly, if 60% or more of the amino acid matches are due to only three individual amino acids, the alignment was considered to be caused by low complexity and was excluded. For the remainder of the alignments, the total length of genes matching transposases was calculated based on the output from the search. If more than 30% of gene length aligned to the transposases, the gene is removed from the gene set. Furthermore, to assess the quality of annotation, *F. vesca* MAKER standard gene set was searched against the Benchmarking Universal Single-Copy Orthologs (BUSCO v.2) plant dataset (embryophyta_odb9)[^21^](https://paperpile.com/c/nMZe3a/nqaV).

To identify novel predicted genes in *F. vesca* v4.1 annotation, *F. vesca* v.2 transcript sequences (Fragaria_vesca_v2.0.a1.transcript.gff3.gz,<https://www.rosaceae.org/species/fragaria_vesca/genome_v2.0.a1>) were extracted from the *F. vesca* v.2 genome using gffread (https://github.com/gpertea/gffread) and mapped to *F. vesca* v.4 genome using MAKER to facilitate the comparison between the two annotations by generating a GFF3 file of *F. vesca* v.2 predicted genes but with *F. vesca* v.4 coordinates. Using BEDTools intersect[^22^](https://paperpile.com/c/nMZe3a/tNoU), *F. vesca* v.4 and *F. vesca* v.2 predicted genes were examined for overlaps and only those genes in *F. vesca* v.4 that have no overlaps were reported. To further filter the set of genes with no overlaps, *F. vesca* v.2 transcript sequences were translated using TransDecoder (http://transdecoder.github.io)(*F. vesca* v.2 protein sequence file is not available in the Rosaceae database) and transcript and protein alignments (TBLASTX, BLASTP, E=10-10) were performed. *F. vesca* v.4 predicted genes that did not have any hits were classified as novel genes. Comparing annotations from different genome assemblies can be challenging and the approach described above has some limitations such that members of a tandem array that are present in the *F. vesca* v4.1 annotation but not in v.2 were possibly filtered out after transcript and protein homology search due to their sequence similarity to other members present in v.2.

**Supplemental Table 3: Gene and feature count and length of F. vesca V4**

| **Feature** | **Count** | **Min. length(bp)** | **Max. length(bp)** | **Mean length (bp)** |
| --- | --- | --- | --- | --- |
| **Gene** | **28,858** | **180** | **47,932** | **1,475** |
| **Exon** | **156,438** | **6** | **7,938** | **152** |
| **Intron** | **127,850** | **4** | **35,125** | **1,334** |

Long terminal repeat retrotransposons (LTR-RTs) were identified using LTR_retriever [^23^](https://paperpile.com/c/nMZe3a/KKlO). In brief, raw LTR-RT candidates identified by LTRharvest [^24^](https://paperpile.com/c/nMZe3a/XY8p) and LTR_FINDER [^25^](https://paperpile.com/c/nMZe3a/45iT) were filtered for the existence of terminal structure (target site duplication and terminal motif) and the matching of LTR-RT Pfam domains. Then the non-redundant LTR-RT library was constructed using the identified intact LTR-RTs for the whole-genome annotation of LTR-RT using RepeatMasker (<http://www.repeatmasker.org/>). Insertion time of intact LTR-RTs were estimated using T=K/2μ, where K is the divergence rate estimated by the Jukes-Cantor model (K=-3/4*ln(1-d*4/3), [^26^](https://paperpile.com/c/nMZe3a/NvKy)) and μ is the neutral mutation rate (1.3 × 10^-8^ mutations per bp per year, [^27^](https://paperpile.com/c/nMZe3a/4JCm)).


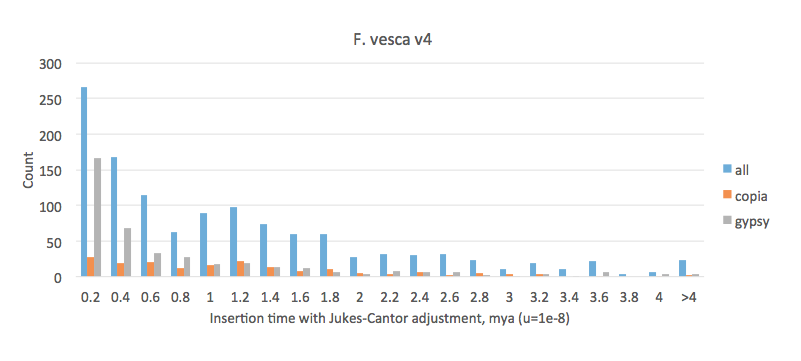


**Supplemental Figure 7: Insertion times of LTR-RTs in the F. vesca V4 genome**

Miniature inverted transposable elements (MITEs) were identified using MITE-Hunter[^28^](https://paperpile.com/c/nMZe3a/zxEj). MITEs were manually checked for TSD and TIR and were classified into superfamilies (*Mutator*, *hAT*, *Tc1Mariner/Stowaway* and *PIF/Harbinger*) but those with ambiguous TSD and TIR were classified as “unknowns.” Using both MITE and LTR library, the *F. vesca* v.4 genome was masked using Repeatmasker and other repetitive elements were identified through Repeatmodeler[^29^](https://paperpile.com/c/nMZe3a/bn5v). The repeats were then categorized into two groups: sequences with and without identities. Those without identities were searched against the transposase database and if they had a match, they were considered a transposon. The repeats were then filtered to exclude gene fragments using ProtExcluder[^12,30,31^](https://paperpile.com/c/nMZe3a/iv1V+gvZB+I4xZ) and an in-house perl script.

**Supplemental Table 4. Transposable elements (TEs) in the *F. vesca* V4 genome.**

| Super-family | | No. of TE homologies | Coverage (Mb) | Fraction of genome (%) |
| --- | --- | --- | --- | --- |
| Class I | LTR/*Copia* | 10267 | 4.43 | 2.01 |
|  | LTR/*Gypsy* | 20055 | 16.45 | 7.47 |
|  | LTR/Unknown | 45417 | 25.25 | 11.46 |
|  | LINE | 9936 | 3.75 | 1.70 |
|  | SINE | 72 | 0.02 | 0.01 |
|  | **Total class I** | 86747 | 49.90 | 22.65 |
| Class II | CACTA | 7347 | 4.86 | 2.21 |
|  | *hAT* | 9940 | 2.95 | 1.34 |
|  | MLE | 318 | 0.08 | 0.04 |
|  | MULE | 14809 | 4.49 | 2.04 |
|  | *PIF/Tourist* | 6760 | 2.05 | 0.93 |
|  | *Helitron* | 1247 | 0.92 | 0.42 |
|  | Unknown | 13217 | 3.12 | 1.42 |
|  | **Total class II** | 53638 | 18.46 | 8.40 |
| **Total TEs** | | 140385 | 68.36 | 31.05 |
| Other unknown repeats | | 42549 | 9.68 | 4.39 |
| **Total repeats** | | 182934 | 78.04 | 35.44 |

**Supplemental Text S5: Organellar Genome Annotation**

The chloroplast genome was annotated using Verdant ([http://verdant.iplantcollaborative.org/plastidDB/index.php#](http://verdant.iplantcollaborative.org/plastidDB/index.php)), a web-based software suite specifically designed for plant chloroplast genomes[^32^](https://paperpile.com/c/nMZe3a/CAw3). Automated annotation of protein coding genes, tRNAs, and rRNAs was completed using annoBTD (<https://github.com/mrmckain/annoBTD>), a novel annotation program designed by the authors of Verdant[^32^](https://paperpile.com/c/nMZe3a/CAw3). Protein-coding regions were found to be variable across different species, so annoBTD program first uses de novo ORF identification to identify potential protein-coding genes. The five most closely related samples in the Verdant database are then selected to be used as a reference for the completion of the annotation. To ensure an accurate annotation, an error corrected *Fragaria vesca* genome was added five times to ensure this correct annotation was used as the reference for the H4 chloroplast genome. The previously identified potential ORFs are BLASTed against the reference genomes using TBLASTX[^33^](https://paperpile.com/c/nMZe3a/KqO7) with an e-value cutoff of 0.1 and a cutoff of 50% identity between references and high scoring segment pairs (HSPs). A references scoring algorithm is then used in case the representative HSP is not complete. Boundaries are refined and identified in the final step of annoBTD. ORF are compared to reference genes using amino acid triplets and if less than 70% of the reference shares triplets with the ORF, it is removed. The best reference for each ORF is used for annotation. An optimized BLASTN[^33^](https://paperpile.com/c/nMZe3a/KqO7) is then used to identify and annotate tRNAs and rRNAs by BLASTing the chloroplast genome against the RNA of the reference genomes. HSP with less than 50% identities are removed and a sliding window of six nucleotides from the reference genome is used to search for RNA in the chloroplast genome. The best scoring references are used to annotate the RNA. Finally, the boundaries of the features are identified based on the sequence and positional information for the features from the chloroplast genome, the sequence information from the best reference for each feature, and the complete sequence of the uploaded genome.


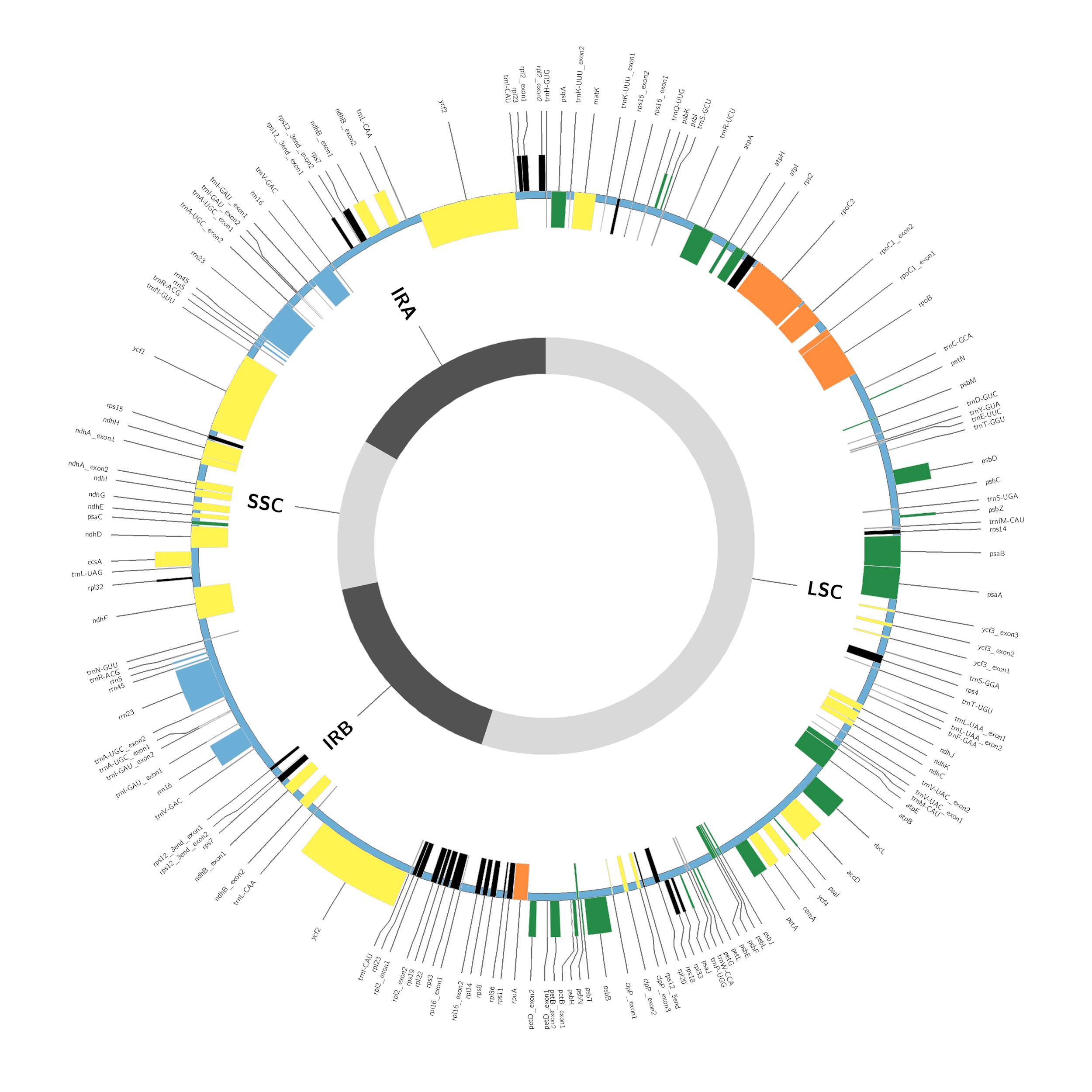


**Supplemental Figure 8: Visualization of the chloroplast genome for *F. vesca* V4.**

The total genome size is 155,640 bp. The assembly was annotated and then visualized using Verdant, a web-based genome annotator for plant chromosomes[^32^](https://paperpile.com/c/nMZe3a/CAw3).

The mitochondrial genome was annotated using the webserver for Mitofy ([http://dogma.ccbb.utexas.edu/mitofy](http://dogma.ccbb.utexas.edu/mitofy.tgz)), a program designed to annotate the genes and tRNAs in the mitochondrial genomes of seed plants[^34^](https://paperpile.com/c/nMZe3a/gQNd). Mitofy uses NCBI-BLASTX to search your genome for genes based in a databases of 41 protein-coding genes found in seed plant mitochondrial plant genomes. The program then uses NCBI-BLASTN to search for tRNAs and rRNAs based on a databases of 27 tRNAs and 3 rRNAs found in plant mitochondrial genome. Mitofy also uses tRNAscan-SE[^35^](https://paperpile.com/c/nMZe3a/4MOW) to identify tRNAs *de novo*. Each annotation was reviewed and the start codon, stop codon, and exon coordinates were manually entered for the annotation. For the mitochondrial genes not found or partially found by Mitofy, the corresponding genes from related species were NCBI-TBLASTN searched against the full H4 mitochondrial genome and the results were used to improve the Mitofy mitochondrial annotation.


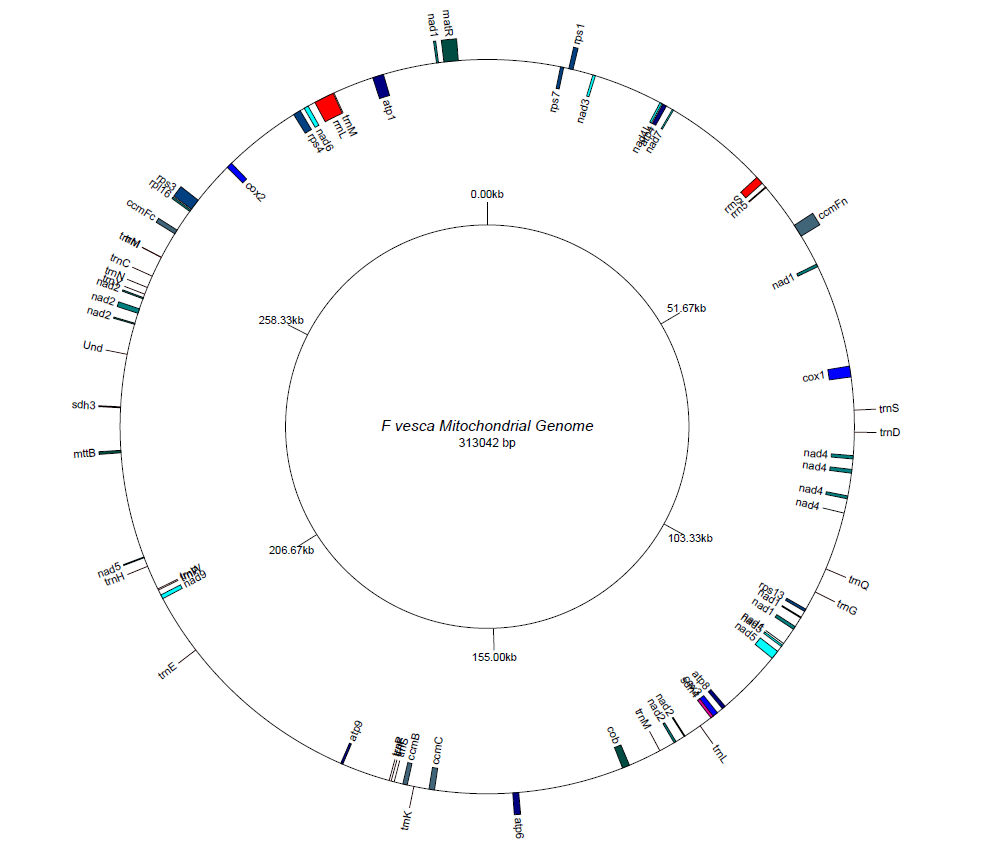


**Supplemental Figure 9: Visualization of the mitochondrial genome for *F. vesca* H4**

The genome size is 313042 bp. The genome was annotated using Mitofy, a program to annotate genes and tRNAs in seed plant mitochondrial genomes[^34^](https://paperpile.com/c/nMZe3a/gQNd).

**Supplemental Text S6: Gene-expression analysis**

Short reads (RNA-seq) from different fruit tissues and developmental stages of *F. vesca* (Table S2) were downloaded from the NCBI-SRA. The SRA files were converted to FASTQ using the NCBI SRATool kit (http://www.ncbi.nlm.nih.gov/Traces/sra/sra.cgi?cmd=show&f=software&m=software&s=software). Illumina adapters were removed from the raw reads using Trimmomatic/0.33[^36^](https://paperpile.com/c/nMZe3a/QNqP) and trimmed reads were filtered using FASTX Toolkit[^37^](https://paperpile.com/c/nMZe3a/9P1O)(http://hannonlab.cshl.edu/fastx_toolkit/index.html). After quality check using FastQC (http://www.bioinformatics.bbsrc.ac.uk/projects/fastqc), the filtered reads were then aligned to the high-quality *F. vesca* genome v.4 using STAR[^38^](https://paperpile.com/c/nMZe3a/M9LC). The reads were then assembled using the reference annotation guided transcript assembly pipeline of StringTie and the output files were converted to read count tables[^16^](https://paperpile.com/c/nMZe3a/X8vC). Gene expression level analysis was performed using DESeq2 pipeline and calculated based on RPKM[^39^](https://paperpile.com/c/nMZe3a/1sho). To visualize expression, 100 genes were randomly selected from the set of novel genes and variance stabilized transformed values were plotted using the pheatmap library in the R statistics package[^40^](https://paperpile.com/c/nMZe3a/jVlN) (**Figure 4**).

**Supplemental Text S7: Synteny and comparative genomics**

Syntenic gene pairs between *F. vesca* V4 (x-axis) and V2 (y-axis)[^41^](https://paperpile.com/c/nMZe3a/fU7I) were identified by DAGChainer[^42^](https://paperpile.com/c/nMZe3a/3FNg), sorted by chromosome (Fvb1-7), and colored based on their synonymous substitution rate as calculated by CodeML[^43^](https://paperpile.com/c/nMZe3a/HipD) using SynMap within CoGe[^44^](https://paperpile.com/c/nMZe3a/sVdc). Syntenic ‘orthologous’ regions are colored in blue and duplicated genes retained from a whole genome triplication event (At-gamma[^45^](https://paperpile.com/c/nMZe3a/NMcd)) in other colors. Pairwise genomic alignments of *F. vesca* V4 and V2 ﬁltered to identify orthologous gene clusters and tandemly duplicated gene copies. Pairs within a physical distance of less than ten genes apart were classiﬁed as tandem duplicate. We identified significantly more tandemly duplicated genes and larger tandem arrays in *F. vesca* V4 (Supplemental Figure 10).


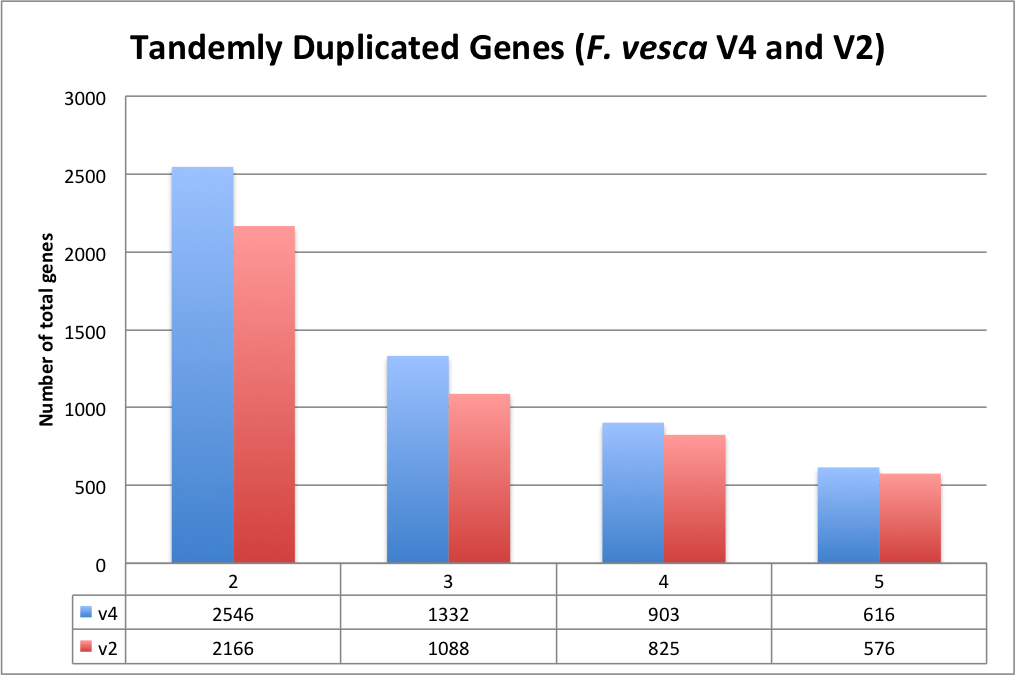


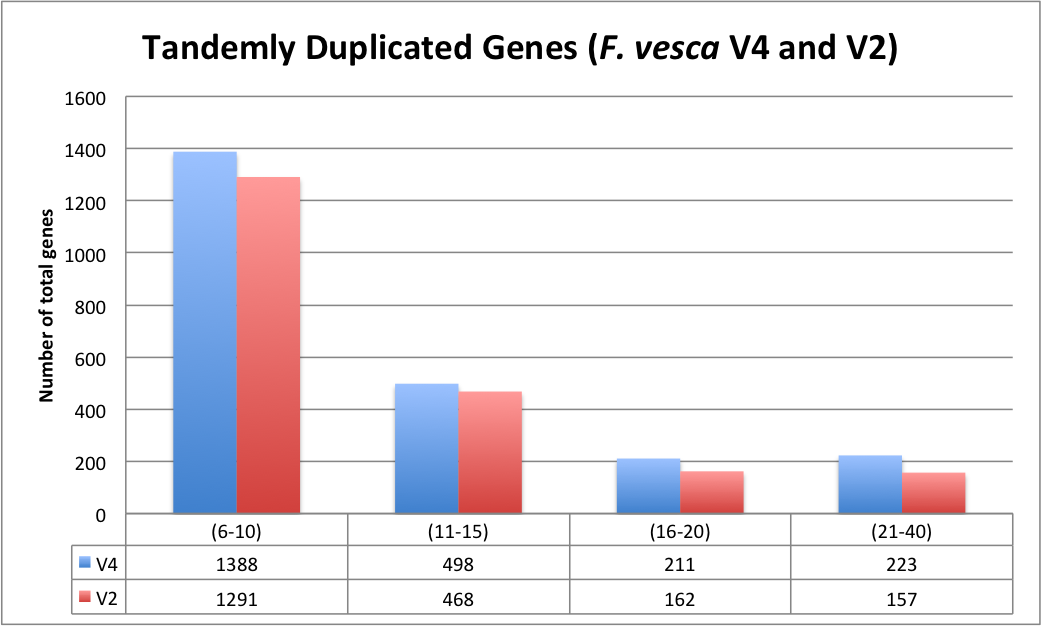


**Supplemental Figure 10: Tandem duplicates identified in *F. vesca* V4 and V2 genomes**

Tandem gene duplicates identified in each version of the genome are plotted by their frequency by array sizes. The top panel includes tandem array sizes ranging from two to five. The bottom panel includes tandem array sizes ranging from 6-10, 11-15, 16-20, and 21-40. The largest array size in V2 is 33 genes and the largest array size in V4 is 39 genes.

**References**

1. [Koren, S. *et al.* Canu: scalable and accurate long-read assembly via adaptive k-mer weighting and repeat separation. *Genome Res.* (2017). doi:](http://paperpile.com/b/nMZe3a/SJEU)[10.1101/gr.215087.116](http://dx.doi.org/10.1101/gr.215087.116)

2. [Chin, C.-S. *et al.* Nonhybrid, finished microbial genome assemblies from long-read SMRT sequencing data. *Nat. Methods* **10,** 563 (2013).](http://paperpile.com/b/nMZe3a/od47)

3. [Walker, B. J. *et al.* Pilon: An Integrated Tool for Comprehensive Microbial Variant Detection and Genome Assembly Improvement. *PLoS One* **9,** (2014).](http://paperpile.com/b/nMZe3a/UePc)

4. [Langmead, B. & Salzberg, S. L. Fast gapped-read alignment with Bowtie 2. *Nat. Methods* **9,** 357–359 (2012).](http://paperpile.com/b/nMZe3a/xZi7)

5. [McKenna, A. *et al.* The Genome Analysis Toolkit: a MapReduce framework for analyzing next-generation DNA sequencing data. *Genome Res.* **20,** 1297–1303 (2010).](http://paperpile.com/b/nMZe3a/I2Px)

6. [Niederhuth, C. E. *et al.* Widespread natural variation of DNA methylation within angiosperms. *Genome Biol.* **17,** 194 (2016).](http://paperpile.com/b/nMZe3a/0VFF)

7. [Schultz, M. D. *et al.* Human body epigenome maps reveal noncanonical DNA methylation variation. *Nature* **523,** 212–216 (2015).](http://paperpile.com/b/nMZe3a/7tde)

8. [Schultz, M. D., Schmitz, R. J. & Ecker, J. R. ‘Leveling’ the playing field for analyses of single-base resolution DNA methylomes. *Trends Genet.* **28,** 583–585 (2012).](http://paperpile.com/b/nMZe3a/xARl)

9. [Wickham, H. *ggplot2: Elegant Graphics for Data Analysis*. (Springer, 2016).](http://paperpile.com/b/nMZe3a/hTrk)

10. [Mahoney, L. L. *et al.* A High-Density Linkage Map of the Ancestral Diploid Strawberry, Fragaria iinumae, Constructed with Single Nucleotide Polymorphism Markers from the IStraw90 Array and Genotyping by Sequencing. *Plant Genome* **9,** (2016).](http://paperpile.com/b/nMZe3a/Ma3n)

11. [Bayer, M. *et al.* Comparative visualization of genetic and physical maps with Strudel. *Bioinformatics* **27,** 1307–1308 (2011).](http://paperpile.com/b/nMZe3a/pHO2)

12. [Campbell, M. S. *et al.* MAKER-P: A Tool Kit for the Rapid Creation, Management, and Quality Control of Plant Genome Annotations. *Plant Physiol.* **164,** 513–524 (2014).](http://paperpile.com/b/nMZe3a/gvZB)

13. [Cheng, C. Y., Krishnakumar, V., Chan, A., Schobel, S. & Town, C. D. Araport11: a complete reannotation of the. *Arabidopsis thaliana* (2016).](http://paperpile.com/b/nMZe3a/bPvX)

14. [Boutet, E., Lieberherr, D., Tognolli, M., Schneider, M. & Bairoch, A. UniProtKB/Swiss-Prot: the manually annotated section of the UniProt KnowledgeBase. *Plant bioinformatics: methods and protocols* 89–112 (2007).](http://paperpile.com/b/nMZe3a/Z4ZF)

15. [Grabherr, M. G. *et al.* Full-length transcriptome assembly from RNA-Seq data without a reference genome. *Nat. Biotechnol.* **29,** 644–652 (2011).](http://paperpile.com/b/nMZe3a/C41p)

16. [Pertea, M. *et al.* StringTie enables improved reconstruction of a transcriptome from RNA-seq reads. *Nat. Biotechnol.* **33,** 290–295 (2015).](http://paperpile.com/b/nMZe3a/X8vC)

17. [Jurka, J. *et al.* Repbase Update, a database of eukaryotic repetitive elements. *Cytogenet. Genome Res.* **110,** 462–467 (2005).](http://paperpile.com/b/nMZe3a/I5uH)

18. [Cantarel, B. L. *et al.* MAKER: an easy-to-use annotation pipeline designed for emerging model organism genomes. *Genome Res.* **18,** 188–196 (2008).](http://paperpile.com/b/nMZe3a/hgnu)

19. [Korf, I. Gene finding in novel genomes. *BMC Bioinformatics* **5,** 59 (2004).](http://paperpile.com/b/nMZe3a/SJOF)

20. [Stanke, M. & Waack, S. Gene prediction with a hidden Markov model and a new intron submodel. *Bioinformatics* **19 Suppl 2,** ii215–25 (2003).](http://paperpile.com/b/nMZe3a/lzML)

21. [Simão, F. A., Waterhouse, R. M., Ioannidis, P., Kriventseva, E. V. & Zdobnov, E. M. BUSCO: assessing genome assembly and annotation completeness with single-copy orthologs. *Bioinformatics* **31,** 3210–3212 (2015).](http://paperpile.com/b/nMZe3a/nqaV)

22. [Quinlan, A. R. & Hall, I. M. BEDTools: a flexible suite of utilities for comparing genomic features. *Bioinformatics* **26,** 841–842 (2010).](http://paperpile.com/b/nMZe3a/tNoU)

23. [Ou, S. & Jiang, N. LTR_retriever: a highly accurate and sensitive program for identification of LTR retrotransposons.](http://paperpile.com/b/nMZe3a/KKlO) In Preparation.

24. [Ellinghaus, D., Kurtz, S. & Willhoeft, U. LTRharvest, an efficient and flexible software for de novo detection of LTR retrotransposons. *BMC Bioinformatics* **9,** 18 (2008).](http://paperpile.com/b/nMZe3a/XY8p)

25. [Xu, Z. & Wang, H. LTR_FINDER: an efficient tool for the prediction of full-length LTR retrotransposons. *Nucleic Acids Res.* **35,** W265–8 (2007).](http://paperpile.com/b/nMZe3a/45iT)

26. [Jukes, T. H. & Cantor, C. R. Evolution of protein molecules Pp. 21--132 in HN Munro, ed. Mammalian protein metabolism. (1969).](http://paperpile.com/b/nMZe3a/NvKy)

27. [Ma, J. & Bennetzen, J. L. Rapid recent growth and divergence of rice nuclear genomes. *Proc. Natl. Acad. Sci. U. S. A.* **101,** 12404–12410 (2004).](http://paperpile.com/b/nMZe3a/4JCm)

28. [Han, Y. & Wessler, S. R. MITE-Hunter: a program for discovering miniature inverted-repeat transposable elements from genomic sequences. *Nucleic Acids Res.* **38,** e199 (2010).](http://paperpile.com/b/nMZe3a/zxEj)

29. [Smit, A. & Hubley, R. RepeatModeler Open-1.0. *Repeat Masker Website* (2010).](http://paperpile.com/b/nMZe3a/bn5v)

30. [Campbell, M. S. Tools and training for genome annotation and analysis. (The University of Utah, 2015).](http://paperpile.com/b/nMZe3a/iv1V)

31. [Campbell, M. S. & Yandell, M. An Introduction to Genome Annotation. *Curr. Protoc. Bioinformatics* **52,** 4.1.1–17 (2015).](http://paperpile.com/b/nMZe3a/I4xZ)

32. [McKain, M. R., Hartsock, R. H., Wohl, M. M. & Kellogg, E. A. Verdant: automated annotation, alignment and phylogenetic analysis of whole chloroplast genomes. *Bioinformatics* **33,** 130–132 (2017).](http://paperpile.com/b/nMZe3a/CAw3)

33. [Camacho, C. *et al.* BLAST+: architecture and applications. *BMC Bioinformatics* **10,** 421 (2009).](http://paperpile.com/b/nMZe3a/KqO7)

34. [Alverson, A. J. *et al.* Insights into the evolution of mitochondrial genome size from complete sequences of Citrullus lanatus and Cucurbita pepo (Cucurbitaceae). *Mol. Biol. Evol.* **27,** 1436–1448 (2010).](http://paperpile.com/b/nMZe3a/gQNd)

35. [Lowe, T. M. & Eddy, S. R. tRNAscan-SE: a program for improved detection of transfer RNA genes in genomic sequence. *Nucleic Acids Res.* **25,** 955–964 (1997).](http://paperpile.com/b/nMZe3a/4MOW)

36. [Bolger, A. M., Lohse, M. & Usadel, B. Trimmomatic: a flexible trimmer for Illumina sequence data. *Bioinformatics* **30,** 2114–2120 (2014).](http://paperpile.com/b/nMZe3a/QNqP)

37. [Gordon, A. & Hannon, G. J. Fastx-toolkit. *FASTQ/A short-reads preprocessing tools (unpublished) http://hannonlab. cshl. edu/fastx_toolkit* (2010).](http://paperpile.com/b/nMZe3a/9P1O)

38. [Dobin, A. & Gingeras, T. R. Mapping RNA-seq Reads with STAR. *Curr. Protoc. Bioinformatics* **51,** 11.14.1–19 (2015).](http://paperpile.com/b/nMZe3a/M9LC)

39. [Love, M. I., Huber, W. & Anders, S. Moderated estimation of fold change and dispersion for RNA-seq data with DESeq2. *Genome Biol.* **15,** 550 (2014).](http://paperpile.com/b/nMZe3a/1sho)

40. [Kolde, R. pheatmap: Pretty Heatmaps. R package version 1.0. 2. (2015).](http://paperpile.com/b/nMZe3a/jVlN)

41. [Tennessen, J. A., Govindarajulu, R., Liston, A. & Ashman, T.-L. Targeted Sequence Capture Provides Insight into Genome Structure and Genetics of Male Sterility in a Gynodioecious Diploid Strawberry, Fragaria vesca ssp bracteata (Rosaceae). *G3* **3,** 1341–1351 (2013).](http://paperpile.com/b/nMZe3a/fU7I)

42. [Haas, B. J., Delcher, A. L., Wortman, J. R. & Salzberg, S. L. DAGchainer: a tool for mining segmental genome duplications and synteny. *Bioinformatics* **20,** 3643–3646 (2004).](http://paperpile.com/b/nMZe3a/3FNg)

43. [Yang, Z. PAML: a program package for phylogenetic analysis by maximum likelihood. *Comput. Appl. Biosci.* **13,** 555–556 (1997).](http://paperpile.com/b/nMZe3a/HipD)

44. [Lyons, E., Pedersen, B., Kane, J. & Freeling, M. The Value of Nonmodel Genomes and an Example Using SynMap Within CoGe to Dissect the Hexaploidy that Predates the Rosids. *Trop. Plant Biol.* **1,** 181–190 (2008).](http://paperpile.com/b/nMZe3a/sVdc)

45. [Bowers, J. E., Chapman, B. A., Rong, J. K. & Paterson, A. H. Unravelling angiosperm genome evolution by phylogenetic analysis of chromosomal duplication events. *Nature* **422,** 433–438 (2003).](http://paperpile.com/b/nMZe3a/NMcd)
