# Supplementary material for: Single-molecule sequencing and optical mapping yields an improved genome of woodland strawberry (Fragaria vesca) with chromosome-scale contiguity
Source: Gigascience. 2017 Dec 13;7(2):gix124. doi: 10.1093/gigascience/gix124 (PMC5801600; doi:10.1093/gigascience/gix124)
Supplement: Response_to_Reviewer_Comments_Original_Submission.pdf [file gix124_response_to_reviewer_comments_original_submission.pdf]

Author's Response To Reviewer Comments

Reviewer #1: Edger P and colleagues present an improved Fragaria vesca genome assembly using PacBio long read sequencing and BioNano optical mapping. In their report, they claimed that their new assembly was one of the most complete and contiguous plant genome assemblies, which is interesting and impressive. In their studies, they compared the new assembly (V4) with the old V2 short read assembly and claimed that they had improved the Fragaria vesca genome assembly to a 'platinum' standard. However, to publish on GigaScience, I think they may address the concerns below:

Response: Thank you for your comments and suggestions. We believe that having addressed these comments helped strengthen the overall quality of the manuscript.

Major:
1. How do authors define 'platinum' quality reference genomes? In what stage can a draft reference genome be called a 'platinum' quality reference genome?

Response: We have changed all instances of ‘platinum quality’ to ‘high-quality’.

2. What was the coverage of the raw 'BspQI' BioNano maps and the coverage of the raw 'BssSI' maps? It will be good to give a statistical report of the raw BioNano maps.

Response: We agree and have added a new table with these details to the supplement.

3. In the manuscript, authors using the 'BspQI' maps completed the first-round hybrid scaffolding and 'BssSI' maps did the second-round hybrid scaffolding. How about changing the enzyme order to perform 'BssSI' hybrid scaffolding first and then the 'BspQI' hybrid scaffolding? Will this change the result and which method gives a better assembly?

Response: The results will be similar no matter which enzyme map is used first, as long as both enzyme produced a high-quality assembly (which is the case here; see the new table in the supplement). Furthermore, the restriction site distribution pattern largely matches between the hybrid scaffolds and the contigs, except for the few instances discussed below. BspQI was chosen for the first round because its assembly was more complete than BssSI (250Mb vs. 214 Mb), and its contiguity was better (2.5 Mb vs. 1.3 Mb).

4. In the first-round BNG hybrid assembly, authors selected the parameter settings as 'cut contig at conflict in BNG maps' and 'cut contig at conflict in NGS sequences'. Shouldn't authors keep the BNG maps and cut the NGS sequences when conflicts occur, as BNG single molecule maps are much longer than the PacBio single reads?

Response: When conflicts occur, the hybrid scaffold algorithm checks the chimeric quality score of the bionano assembly at the break point. If the score >=30, the confidence of single long molecule support of the bionano assembly is normally very strong, and NGS assembly will be cut at this break point. If the score is <30, there might be a chance that the bionano assembly is

chimeric, then the BNG map will be cut. Here, for F. vesca V4, there were 7 cuts made to the contigs and 1 cut made to the BNG map. We manually checked all cut sites, and they all looked quite convincing based on our experience.

5. I noticed that there were still some conflicts between the new V4 assembly and BNG maps. It would be good to validate the BNG hybrid assembly or the final V4 assembly using optical mapping to check how many conflicts unsolved using such as BioNano SV detection (here SV regions should be misassembled regions or conflict regions). What solutions will authors use to solve those detected conflicts?

Response: We (coauthors at BNG) ran Structural Variation (SV) detection between the BspQI assembly and the final V4. There were no major conflicts in the calls with reasonable confidence. 60 deletion calls and 89 insertion calls, all within 150bp, which is less than the optical map resolution range. Our pseudomolecules are also congruent with the published genetic map which we used to anchor the two sets of chromosome arms.

6. How many unknown sequences (gaps) obtained after BNG hybrid scaffolding? How many gaps have been filled in V4 compared to V2? What's the average size of those unfilled gaps? What caused those unfilled gaps?

Response: The F. vesca assembly (Shulaevet al. 2011) has 15,798 contigs with an N50 of 27 kb. The average gap size in the V2 assembly is 1,076 bp. Our assembly has 61 contigs with a contig N50 of 7.9 Mb (before bionano anchoring). Nearly all of the gaps (17Mb of Ns) in the V2 assembly were filled, and our assembly contains ~25 Mb of new sequences. Because this improvement is so drastic and the old assembly had so many erroneous scaffolds, it’s difficult to assess the exact number of gaps that were filled.

37 gaps remained in the V4 assembly after BNG hybrid scaffolding. This includes 23kb of N’s with an average gap size of 621 bp. These gaps likely correspond to highly complex, repetitive regions that are difficult to assemble. These gaps may also include unanchored sequences that had no label sites in the BNG maps.

7. How many predicted genes in the new assembly can be supported by the RNA-seq data or can be supported by the predicted genes in V2? Maybe use a Venn diagram here? What's the reason(s) leading to those unshared genes?

Response: Out of the 28,588 total genes in the annotation, 27,491genes are supported by RNA-seq data. Also, out of the 1496 new genes, 1199 were supported with RNA-seq data. These newly identified genes, not shared in V2, either resided within the gaps in the V2 assembly or were collapsed tandem duplicates.

Minor:
1. In the manuscript, 'previous version' was mentioned several times. I think it is better to specify which version of Fragaria vesca genome assembly was used in the first appearance of the 'previous version'.

Response: We agree. The manuscript has been modified accordingly except instances referencing only new versions of the annotation.

2. I think it is better to use 'the second generation sequencing' to represent the short read sequencing rather than 'the next generation sequencing' (To my knowledge, PacBio sequencing also belongs to the next generation sequencing).

Response: We agree. The manuscript has been modified accordingly.

3. It is better to specify the version of all tools used in the manuscript rather than letting readers find them in the supplementary file.

Response: We have added these details to the manuscript for any tools with multiple versions currently available.

4. It is good to use such as min read length, max read length, average read length and Std to show the stats of PacBio single molecules rather than giving the number of N50. I think N50 is mainly used to show the stats of contigs or scaffolds.

Response: The minimum read length was 3kb (reads shorter than this were filtered prior to assembly) and max read length was 72kb. We sequenced at total of 2,332,270 reads with an average read length of 8,295 bp. For distribution of reads see supplemental Figure 1. We have added these metrics to the manuscript. N50 subread length is commonly used to describe the length distribution of PacBio reads so we have left this in the text.

5. It will be good to specify which method was used to remove chloroplast and mitochondrial genomes? BLAST or others?

Response: We agree - this detail has been added to supplemental methods. BLAST was used to identify the organellar genomes.

***

Reviewer #2: 1. This manuscript provides a beautiful example of lots of existing short-read based genome sequences that need significant improvement so that more authentic biology can be revealed from studies and analysis based on the reference genome sequences.
2. The cost for 80X Pacbio sequence reads, and the optical maps generated by BioNanoGenomic systems could be a hurdle for lots of genome sequencing projects to gain all these kinds of datasets, therefore, new affordable technologies need to be in place in order to improve the quality for all genome sequencing projects.
3. Excited to see that more than 10% new sequences and genes were detected from new data and new assembly.
4. It is very interesting to see that how different genome assemblies affect the profiles of methylation and gene expression, and their effect on the biological explanation of the experiment result.

Response: We really appreciate your positive comments and feedback. In addition, we want to note that the cost of the entire project, both PacBio and BioNano, was under $15,000 USD total. The cost for these genome projects, especially relatively small plant genome projects of this size, are quite affordable now for even a single research programs or between collaborators.
