# Supplementary material for: Single-molecule sequencing and optical mapping yields an improved genome of woodland strawberry (Fragaria vesca) with chromosome-scale contiguity
Source: Gigascience. 2017 Dec 13;7(2):gix124. doi: 10.1093/gigascience/gix124 (PMC5801600; doi:10.1093/gigascience/gix124)
Supplement: Response_to_Reviewer_Comments_Revision_1.pdf [file gix124_response_to_reviewer_comments_revision_1.pdf]

Author's Response To Reviewer Comments

Dear Reviewers and Editor,

Thank you again for all of the helpful comments! The manuscript has been significantly improved thanks to you.

Pat
