# Supplementary material for: Single-molecule sequencing and optical mapping yields an improved genome of woodland strawberry (Fragaria vesca) with chromosome-scale contiguity
Source: Gigascience. 2017 Dec 13;7(2):gix124. doi: 10.1093/gigascience/gix124 (PMC5801600; doi:10.1093/gigascience/gix124)
Supplement: Reviewer_1_Report_(Original_Submission) -- Yuxuan Yuan — 26 Jun 2017 Reviewed [file gix124_reviewer_1_report_(original_submission).pdf]

Reviewer Report

Title: Single-molecule sequencing and optical mapping yields an improved genome of woodland strawberry (Fragaria vesca) with chromosome-scale contiguity

Version: Original Submission **Date:** 6/26/2017

Reviewer name: Yuxuan Yuan

Reviewer Comments to Author:

Edger P and colleagues present an improved Fragaria vesca genome assembly using PacBio long read sequencing and BioNano optical mapping. In their report, they claimed that their new assembly was one of the most complete and contiguous plant genome assemblies, which is interesting and impressive. In their studies, they compared the new assembly (V4) with the old V2 short read assembly and claimed that they had improved the Fragaria vesca genome assembly to a 'platinum' standard. However, to publish on GigaScience, I think they may address the concerns below:Major:1. How do authors define 'platinum' quality reference genomes? In what stage can a draft reference genome be called a 'platinum' quality reference genome?2. What was the coverage of the raw 'BspQI' BioNano maps and the coverage of the raw 'BssSI' maps? It will be good to give a statistical report of the raw BioNano maps.3. In the manuscript, authors using the 'BspQI' maps completed the first-round hybrid scaffolding and 'BssSI' maps did the second-round hybrid scaffolding. How about changing the enzyme order to perform 'BssSI' hybrid scaffolding first and then the 'BspQI' hybrid scaffolding? Will this change the result and which method gives a better assembly?4. In the first-round BNG hybrid assembly, authors selected the parameter settings as 'cut contig at conflict in BNG maps' and 'cut contig at conflict in NGS sequences'. Shouldn't authors keep the BNG maps and cut the NGS sequences when conflicts occur, as BNG single molecule maps are much longer than the PacBio single reads?5. I noticed that there were still some conflicts between the new V4 assembly and BNG maps. It would be good to validate the BNG hybrid assembly or the final V4 assembly using optical mapping to check how many conflicts unsolved using such as BioNano SV detection (here SV regions should be misassembled regions or conflict regions). What solutions will authors use to solve those detected conflicts?6. How many unknown sequences (gaps) obtained after BNG hybrid scaffolding? How many gaps have been filled in V4 compared to V2? What's the average size of those unfilled gaps? What caused those unfilled gaps?7. How many predicted genes in the new assembly can be supported by the RNA-seq data or can be supported by the predicted genes in V2? Maybe use a Venn diagram here? What's the reason(s) leading to those unshared genes?Minor:1. In the manuscript, 'previous version' was mentioned several times. I think it is better to specify which version of Fragaria vesca genome assembly was used in the first appearance of the 'previous version'.2. I think it is better to use 'the second generation sequencing' to represent the short read sequencing rather than 'the next generation sequencing' (To my knowledge, PacBio sequencing also belongs to the next generation sequencing).3. It is better to specify the version of all tools used in the manuscript rather than letting readers find them in the supplementary file.4. It is good to use such as min read length, max read length, average read length and Std to show the stats of PacBio single molecules rather than giving the number of N50. I think N50 is mainly used to show the stats of contigs

or scaffolds.5. It will be good to specify which method was used to remove chloroplast and mitochondrial genomes? BLAST or others?

Level of Interest

Please indicate how interesting you found the manuscript: An article whose findings are important to those with closely related research interests

Quality of Written English

Please indicate the quality of language in the manuscript: Acceptable

Declaration of Competing Interests

Please complete a declaration of competing interests, considering the following questions:

- Have you in the past five years received reimbursements, fees, funding, or salary from an organisation that may in any way gain or lose financially from the publication of this manuscript, either now or in the future?
- Do you hold any stocks or shares in an organisation that may in any way gain or lose financially from the publication of this manuscript, either now or in the future?
- Do you hold or are you currently applying for any patents relating to the content of the manuscript?
- Have you received reimbursements, fees, funding, or salary from an organization that holds or has applied for patents relating to the content of the manuscript?
- Do you have any other financial competing interests?
- Do you have any non-financial competing interests in relation to this paper?

If you can answer no to all of the above, write 'I declare that I have no competing interests' below. If your reply is yes to any, please give details below.

I declare that I have no competing interests

I agree to the open peer review policy of the journal. I understand that my name will be included on my report to the authors and, if the manuscript is accepted for publication, my named report including any attachments I upload will be posted on the website along with the authors' responses. I agree for my report to be made available under an Open Access Creative Commons CC-BY license (http://creativecommons.org/licenses/by/4.0/). I understand that any comments which I do not wish to be included in my named report can be included as confidential comments to the editors, which will not be published.

I agree to the open peer review policy of the journal
